# The antidepressant effect of short- and long-term zinc exposition is partly mediated by P2X7 receptors in male mice

**DOI:** 10.3389/fphar.2023.1241406

**Published:** 2023-10-16

**Authors:** Bernadett Iring-Varga, Mária Baranyi, Flóra Gölöncsér, Pál Tod, Beáta Sperlágh

**Affiliations:** ^1^ Laboratory of Molecular Pharmacology, Institute of Experimental Medicine, Budapest, Hungary; ^2^ János Szentágothai Doctoral School, Semmelweis University, Budapest, Hungary

**Keywords:** purinergic receptor, P2X7, depression, zinc, behavior

## Abstract

**Background:** As a member of the purinergic receptor family, divalent cation-regulated ionotropic P2X7 (P2rx7) plays a role in the pathophysiology of psychiatric disorders. This study aimed to investigate whether the effects of acute zinc administration and long-term zinc deprivation on depression-like behaviors in mice are mediated by P2X7 receptors.

**Methods:** The antidepressant-like effect of elevated zinc level was studied using a single acute intraperitoneal injection in C57BL6/J wild-type and P2rx7 gene-deficient (P2rx7 −/−) young adult and elderly animals in the tail suspension test (TST) and the forced swim test (FST). In the long-term experiments, depression-like behavior caused by zinc deficiency was investigated with the continuous administration of zinc-reduced and control diets for 8 weeks, followed by the same behavioral tests. The actual change in zinc levels owing to the treatments was examined by assaying serum zinc levels. Changes in monoamine and brain-derived neurotrophic factor (BDNF) levels were measured from the hippocampus and prefrontal cortex brain areas by enzyme-linked immunosorbent assay and high-performance liquid chromatography, respectively.

**Results:** A single acute zinc treatment increased the serum zinc level evoked antidepressant-like effect in both genotypes and age groups, except TST in elderly P2rx7 −/− animals, where no significant effect was detected. Likewise, the pro-depressant effect of zinc deprivation was observed in young adult mice in the FST and TST, which was alleviated in the case of the TST in the absence of functional P2X7 receptors. Among elderly mice, no pro-depressant effect was observed in P2rx7 −/− mice in either tests. Treatment and genotype changes in monoamine and BDNF levels were also detected in the hippocampi.

**Conclusion:** Changes in zinc intake were associated with age-related changes in behavior in the TST and FST. The antidepressant-like effect of zinc is partially mediated by the P2X7 receptor.

## 1 Introduction

Major depressive disorder (MDD) is the most common psychiatric disorder, affecting 300 million people worldwide, regardless of age or sex, and causes an extremely high social and economic burden (Trivedi, 2020). A diagnosis of this mental condition requires a substantial mood change lasting at least 2 weeks, such as sadness or irritability, accompanied by a variety of psychophysiological changes, such as decreased sleep or sexual desire, lack of appetite, lethargy, and at its most extreme, suicidal thoughts (Belmaker and Agam, 2008). However, with the development of our knowledge about its pathophysiology and the expanding range of antidepressants, still 29%–46% of patients refuse to take the drugs prescribed by the physician for an inadequate response or at delayed effect (Fava and Davidson, 1996; Schroder et al., 2022). Most antidepressants target the inhibition of transporters responsible for the reuptake of monoamines (Artigas et al., 2002) or stimulation of monoaminergic transmission by other mechanisms (Maes, 1999; Schumacher et al., 2005). In addition, promising research supports the use of the N-methyl-D-aspartate (NMDA) receptor antagonist ketamine in the treatment of MDD and posttraumatic stress disorder (Ates-Alagoz and Adejare, 2013; Sachdeva et al., 2023).

As a complex disease, both genetic and environmental factors play key roles in its development. Several chromosomal regions may be involved in the development of mood disorders (Caspi et al., 2003; Roceri et al., 2004), such as purinergic receptor family member P2X7 (P2rx7) gain-of-function polymorphism (Czamara et al., 2018; Wingo et al., 2021), the role of which in the development of major depression remains controversial (Viikki et al., 2011; Feng et al., 2014). This structure is a non-selective cation channel that belongs to the P2X receptor family, which is sensitive to high ATP concentrations (Sperlagh et al., 2006). It is expressed on many cells of the human body, such as hematopoietic and immune cells, glial cells of the central and peripheral nervous system, central neurons, and hippocampal–cortical pyramidal cells and interneurons. The expression of the P2X7 receptor by neurons remains a subject of longstanding debate (Illes et al., 2017; Miras-Portugal et al., 2017). It modulates neurotransmitter release (Sperlagh et al., 2006; Sperlagh and Illes, 2007), and its activity is attributed to the influx of Ca^2+^ and an increase in the release of glutamate and gamma amino-butyric acid (GABA) from nerve endings (Alloisio et al., 2008) and different areas of the brain (Sperlagh et al., 2002). P2X receptor-mediated currents are modulated by divalent cations, including Zn^2+^ (Acuna-Castillo et al., 2007; Drevets et al., 2022). The receptor-mediated ion current is inhibited through the direct binding site of the extracellular loop (Kasuya et al., 2016) owing to allosteric modulation by agonist binding (Virginio et al., 1997). In terms of the inhibitory effect of divalent metal cations, zinc ranks first in terms of P2rx7 activation. The amino acids involved in this process are histidine and aspartic acid. According to previous measurements, the median inhibition concentration of Zn^2+^ upon activation of mP2X7R is 183 ± 22 µM (Fujiwara et al., 2017).

Several animal experiments on rodents, have demonstrated the involvement of P2rx7 in the pathophysiology of depression. Inhibition of this receptor prevents depression-like behavior in mice (Kongsui et al., 2014; Iwata et al., 2016; Otrokocsi et al., 2017; Ribeiro et al., 2019a; Huang and Tan, 2021), and deletion of the receptor itself results in antidepressant-like behavior (Csolle et al., 2013a). The development of antidepressants that inhibit the P2X7 receptor is currently in the clinical phase (Bhattacharya and Jones, 2018; Drevets et al., 2022) for the treatment of therapy-resistant depression.

Zinc (Zn) is one of the most important essential trace element in the human body and is concentrated in glutamatergic synaptic vesicles. Changes in the extracellular and intracellular concentrations can lead to compromised homeostasis, which can cause or exacerbate psychiatric disorders (Ranjbar et al., 2013; Szewczyk et al., 2018). The hippocampus is the most zinc-rich region of the brain, where high concentrations of zinc are found in the mossy fiber terminals of the CA3 region, which use glutamate as a transmitter (Williams and Undieh, 2010). Zn is released into the extracellular space during neural activity and modulates ion channels (e.g., NMDA and AMPA) that are associated with abnormalities in depression tests. Short-term zinc deficiency causes depression-like behavior as observed in the tail suspension test (TST) and forced swim test (FST) in animals (Whittle et al., 2009; Mlyniec et al., 2012; Mlyniec and Nowak, 2012). Long-term zinc deprivation, lasting 2 weeks, also results in depression-like behavior; however, in this case, measurable differences can be observed in the hippocampal monoamine and zinc content in animals (Tamano et al., 2009), as well as in their serum corticosterone levels (Watanabe et al., 2010). Increased zinc levels as a result of various treatments induce antidepressant-like behavioral patterns in animals, and the immobility time decreases in these tests (Nowak et al., 2003; Szewczyk et al., 2019). Research to measure depression was also carried out in connection with the importance of the age of the animals (Shoji and Miyakawa, 2019), where the FST results may reflect a stronger panic-like response to a sudden aversive stimulus in aged animals, which showed greater immobility than younger mice. In contrast, aged mice showed less immobility than young mice in the TST, suggesting a reduction in depression-related behavior in aged mice. In human studies, serum zinc concentration has been proposed as a potential biomarker for the detection of MDD (Styczen et al., 2017). The concentration of zinc in the serum of patients with MDD was <0.12 μg/mL, whereas that of the control group was 0.66–1.10 μg/mL (Wang et al., 2018). This value fluctuated over a 24-h period; its change reached 20% depending on the food consumed (Roohani et al., 2013).

In this study, we investigated whether the acute administration of zinc in its active form of zinc ion (Zn^2+^) as a ZnCl_2_ solution induces antidepressant-like behavior in the presence or absence of P2X7 receptors. To examine the long-term effect of Zn, mice were fed different Zn-containing controlled diets for 8 weeks. As a result of aging, mice react more sensitively to changes in Zn homeostasis in behavioral experiments. To understand the molecular pathways, we measured monoamine content and brain-derived neurotrophic factor (BDNF) levels in the hippocampus (HC) and prefrontal cortex (PFC). However, zinc-induced antidepressant-like behaviors and changes in behaviors elicited by zinc deprivation in the diet were not consistently eliminated in P2rx7 gene-deficient animals, indicating that the effect of the micronutrient is partially mediated by the receptor.

## 2 Materials and methods

### 2.1 Animals

In this study, behavioral tests and subsequent measurements were performed on 2–3-month “young” (average weight: 30 g) and 16–18-month “elderly” (average weight: 40 g), wild-type P2rx7 +/+ and knockout P2rx7 −/− C57Bl/6J male mice. Preliminary experiments were performed on young 2–3-month-old wild-type and knockout male and female (average weight: 22 g) C57Bl/6J animals. The original breeding pairs of P2rx 7-/- mice were kindly supplied by Christopher Gabel (Pfizer Inc., Groton CT, United States). The breeding and genotyping strategy protocol is described in details in our previous study (Csolle et al., 2013b). All mice in the experiment were housed on a 12 h-on/12 h-off light cycle at controlled temperature (23°C ± 2°C) and humidity (60% ± 10%) in individual plastic cages with *ad libitum* access to food and water. Rodents, such as mice, strongly compete to establish dominance hierarchies in their natural and artificial laboratory environments (Poshivalov, 1980; Capanna et al., 1984). The animals were individually housed to exclude possible confounding effects caused by competition. Preliminary validation experiments indicated that the pre-experiment separation of the animals, although it might have its own effect on behavior, is necessary to reveal consistent antidepressant effects in behavioral tests under our experimental conditions. Experiments and treatments were performed between 9:00 and 13:00 during the light phase (7:00–19:00). All efforts were made to minimize suffering and reduce the number of animals used. All experimental procedures were approved by the local Animal Care Committee of the Institute of Experimental Medicine (Budapest, Hungary, ref. no. PEI/EA/900-7/2020) in accordance with the Institutional Ethical Codex and Hungarian Act of Animal Care and Experimentation guidelines (40/2013, II.14). To use the appropriate number of animals, G*Power 3.1.9.7 software was used to calculate the sample size. For the calculation, we used the main experimental set-up as a basis, thus comparing two groups, WT SAL + WT 1 mg/kg ZnCl_2_, with the following parameters: ANOVA fixed effects, 2-way: *a priori*: compute required sample size; α error probability: 0.05; power: 0.9; effect size: 1.987. Based on these settings, the use of an average of seven animals per group was recommended.

### 2.2 Treatment protocols

**FIGURE 1:**
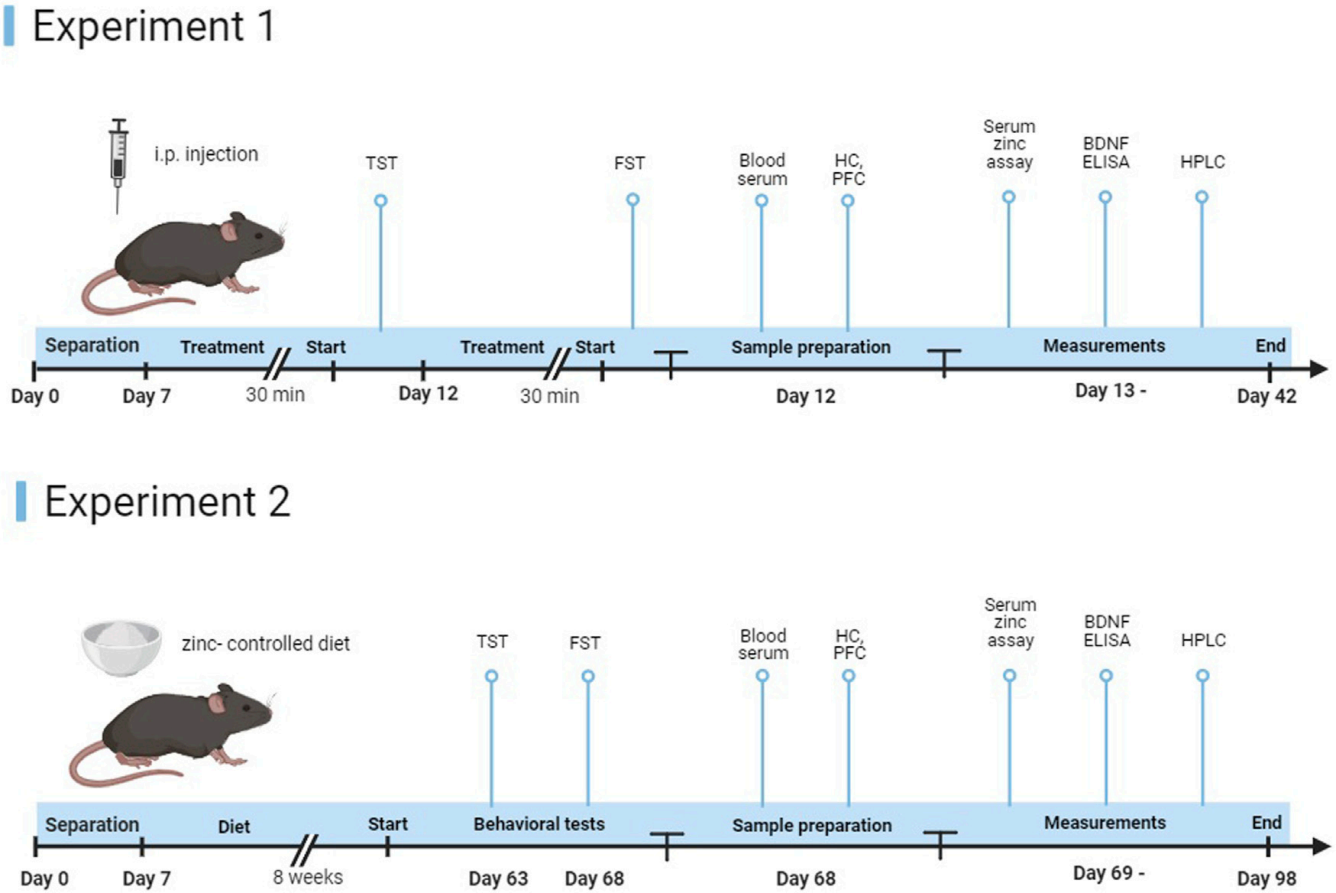
Representation of the study timeline. In experiment 1, young and elderly animals received a single intraperitoneal injection of ZnCl_2_ (1 mg/kg) solution or saline, 30 min before the start of the experiments. After behavioral tests, mice were euthanized (*n* = 8–11 animals/group). In experiment 2, after the 8-week protocol, we started behavioral experiments, followed by euthanasia of the mice (*n* = 8–11 animals/group).

Two different experimental designs were used (Figure 1).

#### 2.2.1 Acute treatment

The first experimental protocol tested the antidepressant effects of a zinc solution. Mice were fed a standard laboratory diet (18 mg M Zn/kg, equivalent to 50 mg/kg zinc sulfate monohydrate, S8189-S095, Ssniff). Prior to each experiment, the animals were treated with an acute intraperitoneal (i.p.) 1 mg/kg ZnCl_2_ (Sigma-Aldrich, United States) solution (0.48 mg M Zn/kg). As a control, physiological saline (0.9% NaCl) was added as previously described. Behavioral experiments (TST and FST) were started 30 min after treatments. The animals were euthanized immediately after the completion of the behavioral tests.

#### 2.2.2 Long-term zinc-controlled diets

To study the long-term effect of zinc on mood-related behavior, in the second experimental protocol, P2rx7 +/+ and −/− mice were fed a higher (23 mg M Zn/kg, which is equivalent to 35 mg/kg zinc-carbonate D10012M, Research Diets) and lower zinc-supplemented and zinc-deficient (4 mg M Zn/kg, did not contain an added source of zinc, D19041002, Research Diets) diet for 8 weeks. Behavioral experiments began after the 2-month period.

### 2.3 Behavioral tests

#### 2.3.1 Tail suspension test

To assess depression-like behavior (Cryan, Mombereau, and Vassout, 2005), experiments were performed using an automated instrument (BIO-TST2, Bioseb, France). The device was connected to a computer that recorded the movement of the animals in real-time. In each trial, three animals were suspended using an adhesive tape placed 1–2 centimeters from the end of their tails. Each chamber was activated 5 s after the last mouse was placed, and the measurements lasted for 6 min. The immobility time during the test was measured in seconds. In some cases, the animals (0%–16%) showed an abnormal movement pattern for the experiment, e.g., climbing on the hook and clinging to the wall of the device. These animals were excluded from the calculations in the *post hoc* analysis.

#### 2.3.2 Forced swim test

The FST series of experiments can be used to detect depression-like phenotypes (Porsolt et al., 1977). The behavioral tests were performed one after the other 5 days apart. Similar to the previous behavioral experiment, the acutely treated animals received an i.p. injection 30 min before the start of the test. The immobility of mice is authoritative when they stop swimming, making movements only to keep their heads above the water surface. The animals were placed in a 2-L (height: 25 cm; diameter: 10 cm) clear glass cylinder (water temperature: 20° ± 2°C) filled to the same water level. The experiment lasted for 6 min for each case. In one study, four animals were simultaneously tested, the water was changed to fresh water, and the rollers were wiped clean before starting a new series of experiments. To prevent the animals from cooling, wipes were placed into their cages to soak up excess water. The results were evaluated using Noldus Observer XT software (Wageningen, Netherlands). The swimming time of the mice was expressed in seconds, and the final value was obtained as a percentage (%) of floating time/experimental time.

### 2.4 Biochemical analyses

#### 2.4.1 Sample preparation

Immediately after the last behavioral experiment, the animals were anesthetized by isoflurane inhalation for blood collection from the inferior vena cava in 2-mL untreated collection tubes. Blood samples were allowed to stand at room temperature (20°C–25°C) for 1 h. Then, they were centrifuged for 15 min at 1,500 x g at 4°C (Megafuge 1.0 R, Heraeus, Germany), and the serum was used for zinc level determination. The samples were stored at −20°C before the assay. After serum collection, the mice were quickly decapitated to remove the HC and PFC. The brain areas were quickly stabilized with liquid nitrogen prior to high-performance liquid chromatography (HPLC) measurements. To measure BDNF protein levels, the prepared brain areas were placed on dry ice and stored at −20°C until homogenization. All samples were used for measurements within 1 month of the euthanization of the experimental animals.

#### 2.4.2 Serum zinc concentration

The frozen samples were thawed at room temperature prior to use. A sensitive fluorometric kit (ab176725, Abcam, United States) was used for quantitative assays. Then, 50 µL of the thawed samples and standard zinc solution was pipetted into a 96-well plate. A detection solution was added to each sample to achieve a final concentration of 100 µL/well. The zinc in the sample bound with high specificity to the zinc detector in the solution, and the zinc probe exhibited a large and greatly increased fluorescence upon exposure to Zn^2+^. We determined the fluorescence increase using a multi-mode plate reader at Ex/Em = 485/525 nm (Cytation™5 Cell Imaging Multi-Mode Reader, BioTek, United States). Zn concentrations (ng/mL) were calculated using GraphPad (GraphPad Software Inc., United States).

#### 2.4.3 Determination of tissue monoamine content

Catechol and indole amines in the tissue extracts were measured by HPLC. The brain tissue homogenate concentration in C57BL/6 and P2X7 receptor-deficient mice was 100 mg/mL. The extract was prepared from hippocampus and prefrontal cortex brain area samples in an ultrasonic homogenizer with 0.1 M perchloric acid (PCA) solution containing theophylline (as an internal standard) at 10 µM concentration and 0.5 mM sodium metabisulphite (antioxidant for biogenic amines). The tissue extract was centrifuged at 3,510 *g* for 10 min at 4°C, and the pellet was saved for protein measurement according to Lowry et al. (1951). Perchloric anions in the supernatant were precipitated using 4 M dipotassium phosphate and removed by centrifugation. Samples were stored at −20°C until analysis, and 10 µL was used for separation. Quantification of the monoamines, noradrenaline (NA), dopamine (DA), and serotonin (5-HT), was performed using an online column switching liquid chromatographic technique. The solid phase extraction was carried out on an HALO Phenyl-Hexyl (75 × 2.1 mm I.D., 5 µm) column, and for separation, it was coupled to an ACE UltraCore SuperC18 (150 × 2.1 mm I.D., 5 µm) analytical column. The flow rate of the mobile phases [“A” 10 mM potassium phosphate, 0.25 mM EDTA “B” with 0.45 mM octane sulphonyl acid sodium salt, 8% acetonitrile (v/v), and 2% methanol (v/v), pH 5.2] was 250 μL/min in a step gradient application (Baranyi et al., 2006). A Shimadzu LC-20 AD HPLC system was used. The signs of the sample components were collected using an Agilent UV (1100 series variable wavelength detector) and a (BAS CC-4) amperometer. Monoamines were electrochemically detected at an oxidation potential of 0.73 V, whereas the internal standard was indicated by UV at 253 nm. Concentrations were calculated using a two-point calibration curve internal standard method: (A_i_ × f × B)/(C × D_i_ × E) (A_i_: area of the biogenic amine component; B: sample volume; C: injection volume; D_i_: response factor of the 1 pmol biogenic amine standard; E: protein content of the sample; f: recovery factor of the internal Standard [IS area in calibration/IS area in actual].

#### 2.4.4 BDNF protein assay

At the beginning of the measurement, the samples were removed from the freezer and weighed. The specimens were processed using a tissue tearor (Model 985370, BioSpec, United States). For homogenization, 250 µL 1-amino-9,10-dihydro-9,10-dioxo-4-[[4-(phenylamino)-3-sulfophenyl]amino]-2-anthracenesulfonic acid sodium salt (PSB) solution and 250 µL lysis buffer (pH = 7.4, 50 mM Tris HCL, 150 mM NaCl, 5 mM CaCl_2_, 0.02% NaN_2_, and 1% Triton X-100 with 0.1% protease inhibitor) were added to each sample. HC and PFC were sonicated at power level 2 using pulses at 1-s intervals for 10–15 s. Subsequently, the samples were centrifuged at 5,000 *g* for 5 min at 4°C. The supernatants were collected and used for the measurements. For the BDNF assays, we used a human/mouse BDNF DuoSet enzyme-linked immunosorbent assay (ELISA) kit (DY248, R&D Systems, United States) and a Pierce™ BCA Protein Assay Kit (23227, Thermo Fisher Scientific, United States). The optical density of the samples (OD) was determined at 450 nm (Cytation™5 Cell Imaging Multi-Mode Reader, BioTek, United States), and the level of BDNF expression (pg/mL) of each sample was calculated against the seven-point standard curve plotted with GraphPad (GraphPad Software Inc, United States). The assay detection limit was 20–1,500 pg/mL. To measure the total protein levels in the samples, absorbance was measured at 560 nm, and values were expressed in pg/mg protein.

### 2.5 Statistics

Statistical analyses were performed using GraphPad Prism software v.8.0.2. (GraphPad Software Inc., United States). All data were presented as the mean ± SEM of “n” determinations. In behavioral experiments, animals with incorrect movements were excluded from the analysis. Other possible outlier values were detected by the ROUT method (q = 1%) (Motulsky and Brown, 2006). Data from behavioral tests and biochemistry analyses were analyzed by two- and three-way ANOVA, followed by Tukey’s *post hoc* test. For determination of monoamine content, data were expressed as the mean ± standard error of mean at pmol/mg protein concentration. Data processing, calculations, and graphical representation were performed using Microsoft Office Excel 2010, and the TIBCO Data Science Workbench was used for statistical analysis. The Kolmogorov–Smirnov test was used to examine the normality of all continuous variables in the measurement. Where the measured variables met the normality assumption, factorial analysis of variance (FR-ANOVA) was used. We determined group differences in HC and PFC monoamine variance caused by acute i.p. zinc administration and long-term feeding with zinc-supplemented and -deficient diets in young and elderly, wild-type and P2X7 receptor-deficient mice. The threshold for statistical significance was set at *p* < 0.05. For detailed statistical tests of all experiments, the n and *p*-values are given in Supplementary Table S1 **.**

## 3 Results

### 3.1 The antidepressant effect of a single intraperitoneal ZnCl_2_ treatment is not associated with the P2X7 receptor

In the acute animal studies, we hypothesized that a single intraperitoneal ZnCl_2_ treatment would exert antidepressant effects. Preliminary experiments (30, 10, 3, 1, and 0.5 mg/kg ZnCl_2_, converted: 14.39, 4.8, 1.44, and 0.24 mg M Zn/kg) were performed to determine the dose (Supplementary Figures S1A–E). Higher doses caused more toxic effects; therefore, 1 mg/kg was selected for subsequent experiments (Supplementary Figures S1. F). To assess whether acute inhibition of P2rx7 with zinc can modify locomotor behavior, we performed an open field test (OFT) (Supplementary Material S1) on young, acutely treated male mice. ZnCl_2_ treatment did not affect the activity of wild-type and P2X7 KO mice compared to saline-treated groups, and there was no significant difference in the locomotion of WT and P2X7 KO mice after i.p. administration (Supplementary Figure S2).

**FIGURE 2:**
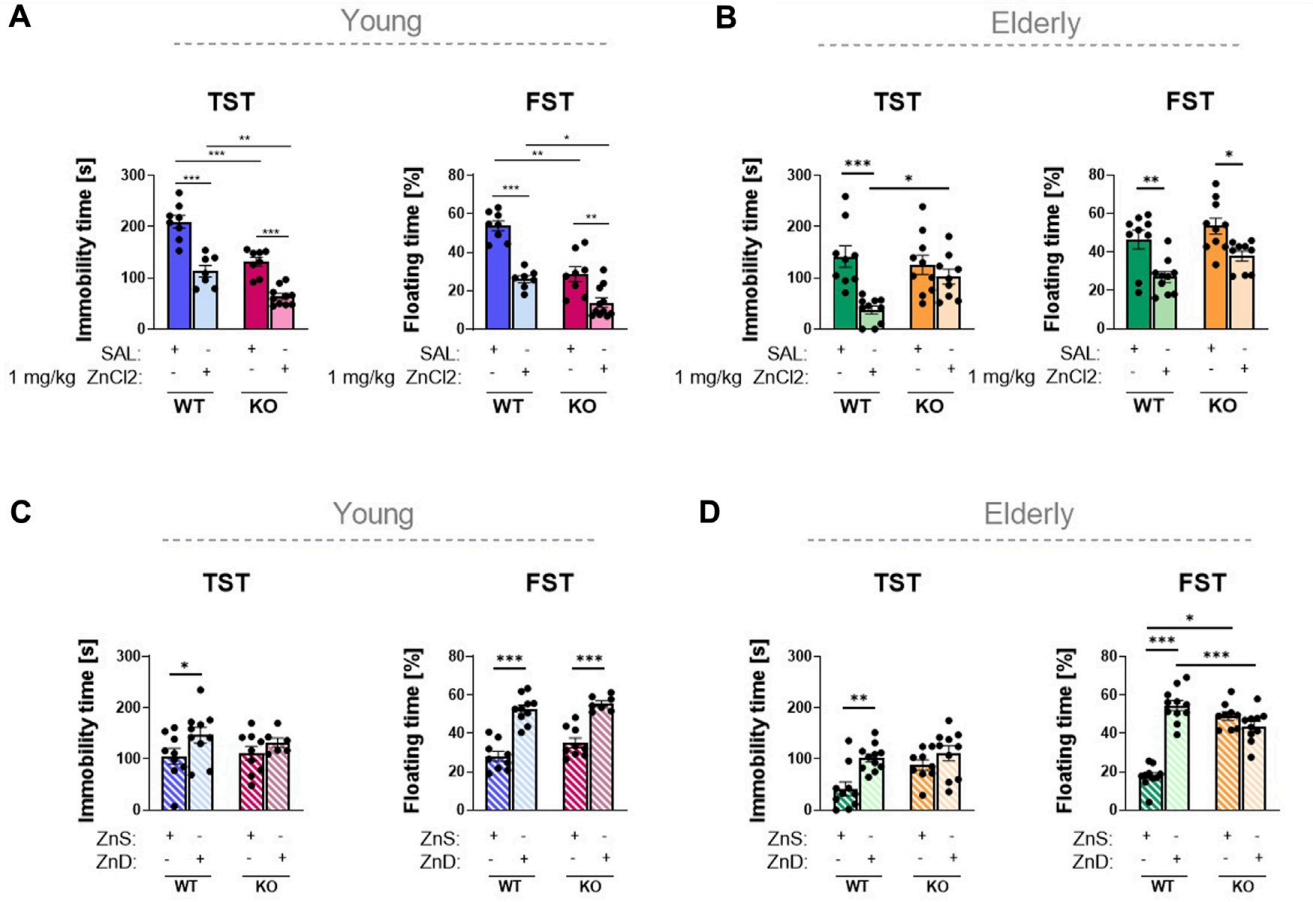
Investigation of the association between zinc content and depression in animal experiments **(A–D)**. P2rx7 +/+ control or P2rx7 −/− mice were treated with a single intraperitoneal injection of 1 mg/kg ZnCl_2_ or saline in young (*n* = 7–11) **(A)** and elderly (*n* = 9–10) **(B)** animals. In the long-term experiments, wild-type or P2rx7-KO young (*n* = 6–10) **(C)** and elderly (*n* = 9–11) **(D)** animals were fed with a Zn-controlled diet. TST and FST behavior experiments were performed, the immobility or floating time is shown in the bar diagrams. Data are expressed as mean ± S.E.M. Data were analyzed by two-way ANOVA, followed by Tukey’s test. *p < 0.05; **p < 0.01; ***p < 0.001. TST: tail suspension test; FST: forced swim test; WT: wild-type; KO: knockout; ZnS: zinc-supplemented diet; ZnD: zinc-deficient diet.

Confirming the results described in previous studies (Basso et al., 2009; Csolle et al., 2013a), P2rx7 deficiency elicited an antidepressant-like effect in male mice (Figure 2A). As a result of 1 mg/kg zinc solution, both the immobility and floating time of the mice in TST and FST, respectively, were substantially reduced in P2rx7 +/+ young adult animals. These effects were replicated in P2rx7 −/− mice, suggesting that the antidepressant-like effect of acute zinc treatment was negligibly mediated by P2X7 receptors (Figure 2A). We tested the antidepressant-like effects of zinc in young adult female mice. No differences were observed between the saline-treated wild-type and P2rx7-deficient animals. Moreover, 1 mg/kg zinc administration substantially increased the immobility time of wild-type animals in the TST, but not in the FST. No effects of acute zinc treatment were observed in female P2rx7-deficient mice (Supplementary Figure S3).

Identical experiments were performed using elderly male mice. No significant differences were observed among the genotypes of these animals (Figure 2B). In the TST, zinc treatment caused a decrease in the immobility time in wild-type animals; however, in P2rx7-deficient animals, zinc did not have a significant effect. In the FST, zinc treatment substantially decreased the immobility of both P2rx7 +/+ and P2rx7 −/− mice, with an overall genotype effect, but without a significant interaction. Because the effect of the treatment was also observed in knockout animals, it can be concluded that besides the TST in elderly animals, the effect of a single ZnCl_2_ injection is not mediated by the P2X7 receptor.

**FIGURE 3:**
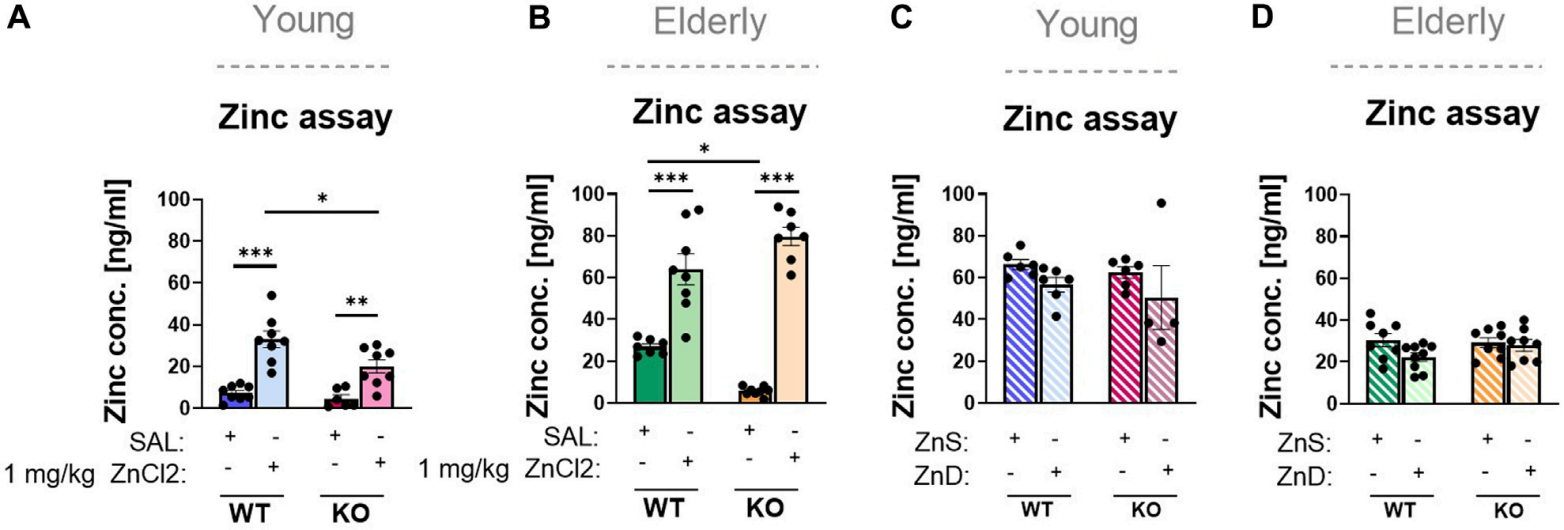
Results of zinc content in blood serum **(A–D)**. Wild-type and knock out, young (*n* = 6–8) **(A)** and elderly (*n* = 7–8) **(B)** mice were injected with an acute treatment of ZnCl_2_ (1 mg/kg) or saline. P2rx7 +/+ and −/−, young (*n* = 4–6) **(C)** and elderly (n = 8–9) **(D)** mice were fed a Zn-controlled diet. Data are expressed as mean ± S.E.M. Data were analyzed by two-way ANOVA, followed by Tukey’s test. *p < 0.05; **p < 0.01; ***p < 0.001. WT: wild-type; KO: knockout; ZnS: zinc-supplemented diet; ZnD: zinc-deficient diet.

To verify the effect of acute zinc injection, we measured serum zinc concentrations in the blood after the injections, which displayed substantial elevations in both young adults and elderly mice of both genotypes (Figures 3A, B).

### 3.2 The zinc-deficient diet caused depression-like behavior in young and elderly animals, which was partly mediated by the P2X7 receptor

Next, we compared the effect of a zinc-containing diet over a 2-month period on the behavior of male mice with no zinc supplementation. As a result of the long-term experiments, relative zinc deficiency induced depression-like behavior in young mice, as reflected in longer immobility time in both TST and FST (Figure 2C). In contrast, no significant difference was detected in knockout animals that received either zinc-enriched or zinc-deprived diets in the TST, but not in the FST, indicating that the relative depression-like effect of the zinc-deficient diet might be partly mediated by P2X7 receptors. Similar results were observed in the elderly group that received different zinc-containing diets (*n* = 9–11 animals/group) (Figure 2D). In the wild-type groups receiving the zinc-deficient diet, substantially higher values of immobility were measured in both the TST and FST. We did not observe any significant difference in the weights of the animals during the 8-week experiment (Supplementary Figure S4). Just like in young adult mice, the depression-like effect of zinc deficiency was eliminated in the absence of the P2X7 receptor in the elderly groups.

Despite the different Zn^2+^ content of the diet, serum Zn^2+^ levels were uniformly increased when compared to those of the animals kept on a standard laboratory diet, probably because of the difference between the two diets in overall Zn ^2+^ content (Figures 3C, D). This was true for both young adult and elderly animals, although the serum zinc levels were considerably lower in the latter group (Figure 3D). In summary, we observed that in the case of the TST in young adult and in elderly mice, the antidepressant-like effect of long-term zinc enrichment was partially P2X7 receptor-dependent.

### 3.3 Excess zinc intake increased the serotonin content of the hippocampus

**FIGURE 4:**
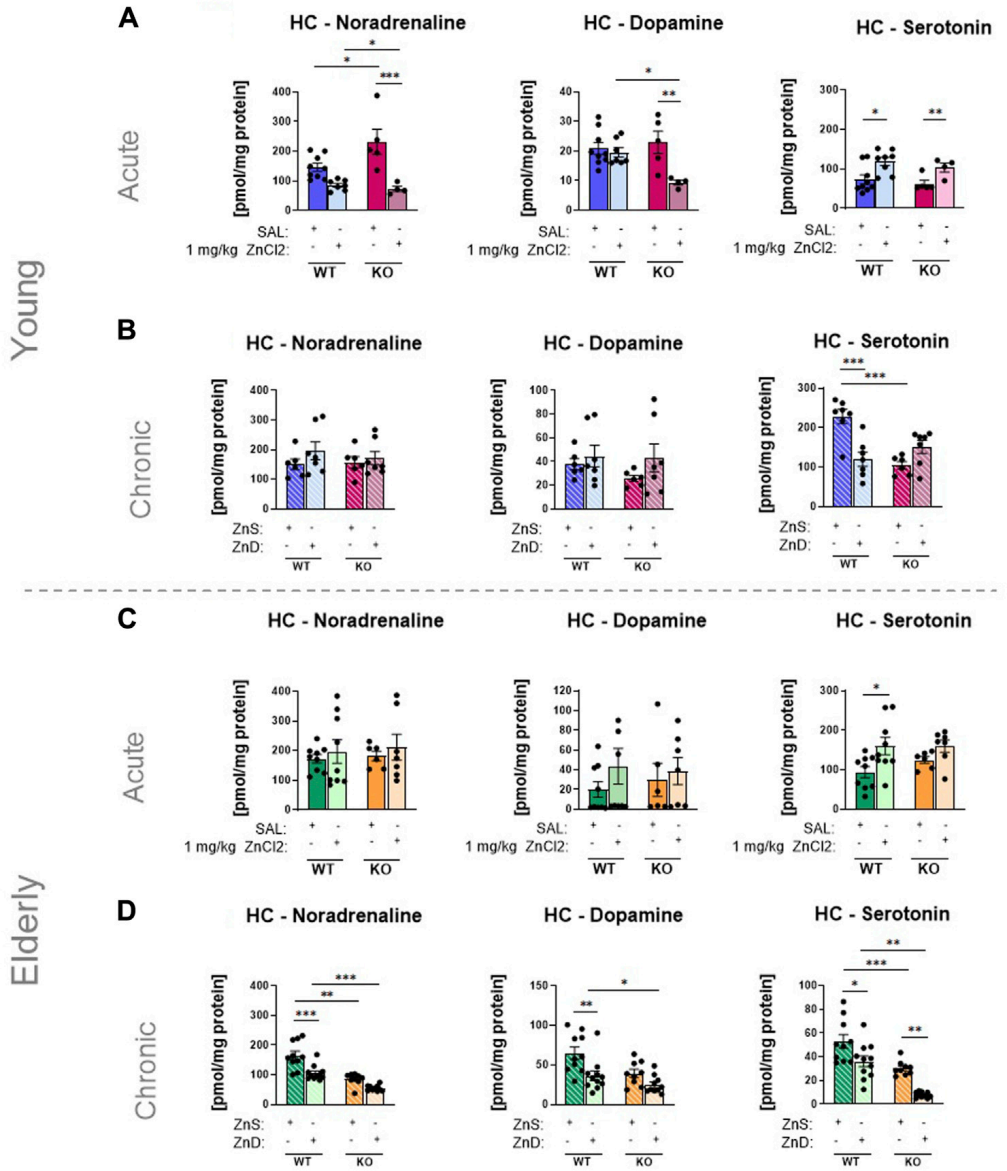
Zinc intake affects the monoamine contents of the hippocampus **(A–D)** using HPLC technique. Noradrenaline, dopamine and serotonin levels in P2rx7 +/+ and P2rx7 −/− mice were plotted in the acute (*n* = 5–9) **(A)** and long-term fed with zinc-controlled diets young (*n* = 6–7) **(B)** animals. Monoamine contents were also measured in elderly animals, that received similar acute treatment (n = 6–9) **(C)** or a zinc-controlled diet (*n* = 9–11) **(D)**. Data are expressed as mean ± S.E.M. Data were analyzed by two-way ANOVA followed by Tukey’s test. *p < 0.05; **p < 0.01; ***p < 0.001. HC: Hippocampus; WT: wild-type; KO: knock out; ZnS: zinc-supplemented diet; ZnD: zinc-deficient diet.

After the behavioral experiments, we examined how our results could be explained by two accepted theories of depression development: the monoamine and BDNF hypotheses. Using HPLC, concentrations of monoamines, such as NA, DA, and 5-HT, were measured in the HC, a brain area relevant to the behaviors measured in the FST and TST (Hao et al., 2019). Following a single injection of ZnCl_2_ solution, 5-HT levels in the HC of young mice were increased in both wild-type and knockout animals (Figure 4A), whereas no treatment-induced changes were observed in DA and NA levels (Figures 4A–D). Likewise, a long-term zinc-deficient diet decreased serotonin levels in the HC (Figure 4B), but only in wild-type mice, which was consistent with the behavioral results found in the TST (Figure 2C). In the elderly group, a substantial increase in 5-HT levels was observed in P2rx7 +/+ mice, but not in KO mice, which was consistent with the results of the behavioral experiment (Figures 2B, 4C). In contrast, the zinc-deficient diet reduced the levels of all three monoamines in elderly wild-type mice (Figure 4D), which might be associated with lower basal serum zinc levels in these animals (Figure 3D). In the PFC, we detected treatment-related changes in monoamine levels only in the elderly animals (Supplementary Figures S5A, B). The 5-HT content increased in the wild-type group, but not in the P2rx7 −/− animals acutely treated with ZnCl_2_ (*n* = 6–7 animals/group) (Supplementary Figure S5A). In the elderly group, long-term feeding of zinc-deprived diet animals (*n* = 9–11 animals/group), NA and 5-HT levels were reduced in a P2X7-dependent way in the PFC (Supplementary Figure S5B).

**FIGURE 5:**
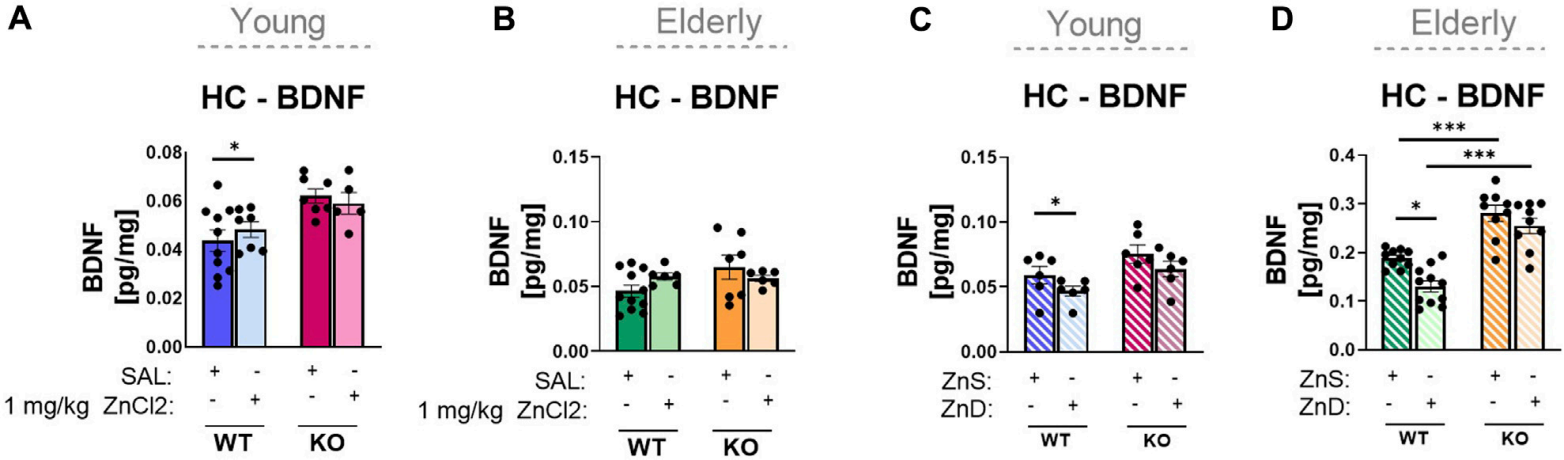
Examination of hippocampus BDNF protein content by ELISA **(A–D)**. Wild-type and knockout young (*n* = 5–11) **(A)** and elderly (*n* = 6–11) **(B)** mice were injected intraperitoneally with an acute treatment of saline or ZnCl_2_ (1 mg/kg). P2rx7 +/+ and −/− young (*n* = 6) **(C)** or elderly mice were fed a Zn-controlled diet (*n* = 9–11) **(D)**. Data are expressed as mean ± S.E.M. Data were analyzed by two-way ANOVA, followed by Tukey’s test. *p < 0.05; **p < 0.01; *** *p* < 0.001. HC: hippocampus; WT: wild-type; KO: knockout; ZnS: zinc-supplemented diet; ZnD: zinc-deficient diet.

Another important neurochemical readout of an antidepressant action is the change in hippocampal BDNF levels. Corresponding to the effects detected in behavioral studies, in young adult animals, acute ZnCl_2_ treatment increased, where Zn deprivation reduced hippocampal BDNF levels, with an overall genotype effect in the former group, but without interaction (Figures 5A, C). In the elderly group, acute ZnCl_2_ treatment did not affect the BDNF level in the hippocampus of wild-type animals (Figure 5B), whereas zinc-controlled diet and Zn deprivation decreased it (Figure 5D). In P2rx7-deficient animals, neither acute ZnCl_2_ treatment nor zinc deprivation had any effect on hippocampal BDNF levels in either age groups (Figures 5A–D). Similar data were obtained during the PFC examination (Supplementary Figure S6). Acute zinc treatment caused an increase in BDNF levels only in young P2X7 +/+ animals (Supplementary Figure S6A). The long-term diets showed changes between genotypes in the elderly group, and both diets increased the BDNF levels in the knockout males (Supplementary Figure S6D).

## 4 Discussion

The antidepressant action of P2rx7 inhibition (Csolle et al., 2013b; Wang et al., 2016; Ribeiro et al., 2019b) and Zn^2+^ (Mlyniec et al., 2012; Mlyniec et al., 2014; Wang et al., 2018) has been well-established in animal studies, and the inhibitory action of Zn^2+^ on P2X7 receptors has also been documented (Moore and Mackenzie, 2008; Kovacs et al., 2018; Martinez-Cuesta et al., 2020). Zn^2+^ and other divalent cations inhibit P2rx7-mediated ion currents via direct binding to the extracellular loop (Liu et al., 2008; Kasuya et al., 2016). Zn is a micronutrient essential for the functioning of the human body and predominantly accumulates in specific regions of the brain, such as the cortex, hippocampus, and amygdala. The largest amount is bound to metalloproteins; the rest is stored in presynaptic vesicles, and the neurons containing them are called zinc-enriched neurons (ZENs) (Takeda, 2000). In the cortex and hippocampus, these ZEN terminals are associated with glutamatergic neurotransmission (Westbrook and Mayer, 1987), whereas in the cerebellum, they are associated with GABA neurotransmission (Wang et al., 2002). By inhibiting glutamate and GABA receptors, zinc plays an important role in synaptic plasticity (Wolf et al., 2018) and can modify the excitability of neurons (Sperlagh et al., 2002; Acuna-Castillo et al., 2007). Zn also inhibits P2X7-mediated functional responses in the hippocampus, such as increased glutamate release in acute slices (Sperlagh et al., 2002) and propidium uptake by hippocampal astrocytes (Kovacs et al., 2018).

Based on these data, our primary goal was to investigate whether P2rx7 plays a role in the mechanism of action of zinc in modulating depression-like behavior in mice in the TST and FST. Simple behavioral tests are widely used for screening potential antidepressant effects; however, they have some drawbacks. Certain drugs and antidepressants increase motor activity, thus yielding false positive results in the aforementioned behavioral experiments (Borsini and Meli, 1988). To achieve this goal, we examined the effects of acute systemic ZnCl_2_ treatment and a special diet enriched or deprived of zinc. To examine these two effects, we used diets with different zinc contents for the acute and long-term zinc-regulated series of experiments. In the acute protocol, we investigated the antidepressant effects of a single excess dose of Zn. In this case, the diet of the mice did not change compared with the previously used standard laboratory diet, and we measured the changes caused by the injected zinc solution. In the 8-week zinc-controlled diet series of experiments, we examined depression-like behavior caused by zinc deficiency, where we built our own protocol based on literature data (Mlyniec et al., 2012). To validate the effect of the aforementioned treatments, zinc concentrations were measured in the serum following these interventions as based on a previous study, where serum and brain zinc levels were inferred from each other (Wang et al., 2010). In accordance with the available literature (Rafalo-Ulinska et al., 2022), the serum zinc concentration in wild-type mice was substantially increased by acute zinc treatment. Rafało-Ulińska et al. administered 40 mg zinc hydroaspartate/kg or 0.9% NaCl solution orally to their male mice, 1 h before decapitation. Based on their measurements, compared to the serum zinc concentration of the control animals (0.4 ± 0.04 μg/mL), the zinc content of the blood serum of the treated animals was 15 times higher, 6.0 ± 0.85 μg/mL. In our experiments, the 1 mg/kg ZnCl_2_ solution with the shorter duration of action (30 min), compared to the 0.9% NaCl i.p., compared to treated control mice (7.29 ± 1.27 ng/mL), resulted in a nearly 5-fold higher serum zinc concentration (33.05 ± 4.02 ng/mL). A similar change was observed in the P2rx7 −/− animals; compared to the serum zinc concentration of 4.69 ± 1.77 ng/mL in the controls, we measured a value of 20.03 ± 3.17 ng/mL in the treated mice. Accordingly, acute zinc intake in young and elderly animals reduced the floating time for both genotypes in the behavioral experiments, except for the TST of old animals, where no significant difference was found in the immobility time of the KO animals. This is an antidepressant-like effect, which cannot be explained by the action on locomotor activity, as we could not find either a treatment- or genotype-related difference between the groups in the open field arena within the timeframe of the FST and TST (Supplementary Figure S2). We also replicate previous literature data showing that P2rx7 deficiency by itself exhibited an antidepressant effect, and regarding the movement of the control, that of WT mice was identical to the results of previous experiments (Kroczka et al., 2000; Szewczyk et al., 2002; Nowak et al., 2003; Rosa et al., 2003; Csolle et al., 2013a). However, we were not able to replicate these results in young female mice. One possible explanation for these findings is that the estrous cycle influences the responsiveness of female animals to these tests. The estrous cycle may have an effect on female mouse behavior in anxiety- and fear-related tests compared to male mice (Lovick and Zangrossi, 2021). We also failed to observe an antidepressant-like effect of zinc in female mice, which is noteworthy, as previous studies on the association between zinc and depression were also performed in male animals (Mlyniec et al., 2012; Mlyniec and Nowak, 2012). Therefore, because the prevalence of depression is higher in female animals, the effects of excess zinc and zinc deprivation on depression-like behaviors in female mice require further investigation. Our results in male mice imply that the antidepressant-like effect of acute systemic ZnCl_2_ treatment is largely independent of the genotype and is mediated by other signaling pathways, such as glutamatergic or GABAergic transmission, or a direct or indirect impact on BDNF levels, as confirmed in the present study (Figure 5A). When animals were fed a long-term, Zn-controlled diet, serum zinc levels were higher than those in the animals fed a standard laboratory diet irrespective of the genotype, in young adults but not in elderly animals. The exact reason for this discrepancy is unknown; however, the most likely reason is the different actual zinc content of the standard laboratory diet (18 mg M Zn/kg) and the Zn-controlled diet (23 mg M Zn/kg). Zinc deprivation in the controlled diet group did not affect serum zinc concentrations. This is consistent with the findings of Wong et al. (2009) and might be explained by the fact that, in contrast to the acute, high-dose treatment, fewer micronutrients were administered to the mice for a longer period, so the serum levels could be saturated.

We have also found differences in basal immobility values between the experiments assessing the effect of acute Zn treatment and the effects of the zinc-controlled diet. The difference in the results obtained by the control groups in the behavioral tests can be explained by the different experimental designs. The mice in the zinc-controlled diet experiment did not receive an injection of saline solution before the behavioral test, which itself is a stressful stimulus. This assumption is supported by the fact that the basal immobility time of wild-type mice is uniformly lower in long-term zinc-regulated diet experiments with naïve mice in both the young adult and elderly groups than in their acutely saline-treated counterparts.

Nevertheless, in our experiments, we showed that a long-term zinc-deprived diet increased immobility in both the FST and TST, and the effect in the TST was attenuated in P2rx7-deficient mice. The same effects were observed in elderly animals, that is, the pro-depressant effect of zinc deprivation was also lost in the absence of P2X7Rs. These results indicate that, in the case of an acute Zn^2+^ treatment, the divalent cation has a multiplicity of molecular targets, especially ion channels that might mediate its effect on depressive-like behavior. These include NMDA, α-amino-3-hydroxy-5-methyl-4-isoxazolepropionic acid (AMPA), GABA receptors, Zn transporters, and G protein-coupled receptor 39 (GPR39) (Costa et al., 2023). The immobility time of wild-type control animals of the same age, but participating in different experiments, varied more than that of P2X7 KO mice (Figures 2A, B). Further experiments are required to determine the specific explanation; however, according to our observations and those of others (Smith et al., 2020), in general, P2X7 KO animals are calmer than their peers and adapt more easily to changing conditions. This is supported by the attenuated adrenocorticotropic hormone and corticosterone responses to acute stress (Csolle et al., 2013b; von Muecke-Heim et al., 2021). In addition, P2X7 receptors might participate in the effect of the TST in old animals; however, this partial involvement argues against the major contribution of the P2X7 receptor to the antidepressant effect of an acute, systemic Zn load. In contrast, in the case of more subtle, long-term changes in Zn concentrations in the local brain microenvironment caused by dietary changes, the inhibitory effect of Zn on P2X7 receptor channels might be more substantial as a mediator of Zn^2+^-related actions on behavior, as observed in the TST in both young adult and elderly animals and in the corresponding neurochemical alterations.

While searching for potential mechanisms of action, we examined brain monoamine and BDNF levels, which are consistent with the underlying hypotheses of depression-like behaviors. According to the monoamine hypothesis, the pathophysiological basis of depression is a deficit in monoaminergic transmission in the central nervous system (Delgado, 2000; Hirschfeld, 2000), whereas the BDNF theory emphasizes that depression is owing to dysfunctional neurogenesis in brain regions responsible for emotions and cognition (Duman and Monteggia, 2006); that is, the expression of neuronal growth factors decreases when we perceive a stress effect (Kim et al., 2020). In our experiments, as a result of acute Zn injection, the concentration of 5-HT in the HC was increased in both young and old mice, all of which validated the results of the behavioral experiments. Moreover, long-term Zn deprivation reduced monoamine levels in both age groups, indicating that the hippocampal NA, DA, and 5-HT levels of elderly animals are more sensitive to treatment. In this group, changes in PFC monoamine levels were also consistent with depression-like behavior caused by the zinc-deprived diet, especially in the case of NA and 5-HT, which are key pharmacological targets in mood disorders. As for BDNF measurements, our data partially support the results found in the literature, demonstrating increased hippocampal BDNF levels in young adult wild-type animals in response to the acute Zn treatment and a decrease in response to Zn deprivation (Williams and Undieh, 2010; Csolle et al., 2013a; Starowicz et al., 2019; Rafalo-Ulinska et al., 2020). The effect of acute Zn treatment to stimulate BDNF production in young adult wild-type mice is also demonstrated in the PFC. However, in old animals, only zinc deprivation, but not acute treatment, affected BDNF levels and only in wild-type animals.

In conclusion, our data demonstrate that the antidepressant-like effect of extracellular zinc is partially mediated by P2rx7 in a male mouse model, which is supported by changes observed in behavioral experiments. The extent of its contribution probably also depends on the age of the animal and the initial zinc saturation in the serum and brain.

## Data Availability

The original contributions presented in the study are included in the article/Supplementary Material; further inquiries can be directed to the corresponding author.
