# Supplementary figures and images for: The antidepressant effect of short- and long-term zinc exposition is partly mediated by P2X7 receptors in male mice
Source: Front Pharmacol. 2023 Oct 16;14:1241406. doi: 10.3389/fphar.2023.1241406 (PMC10613712; doi:10.3389/fphar.2023.1241406)

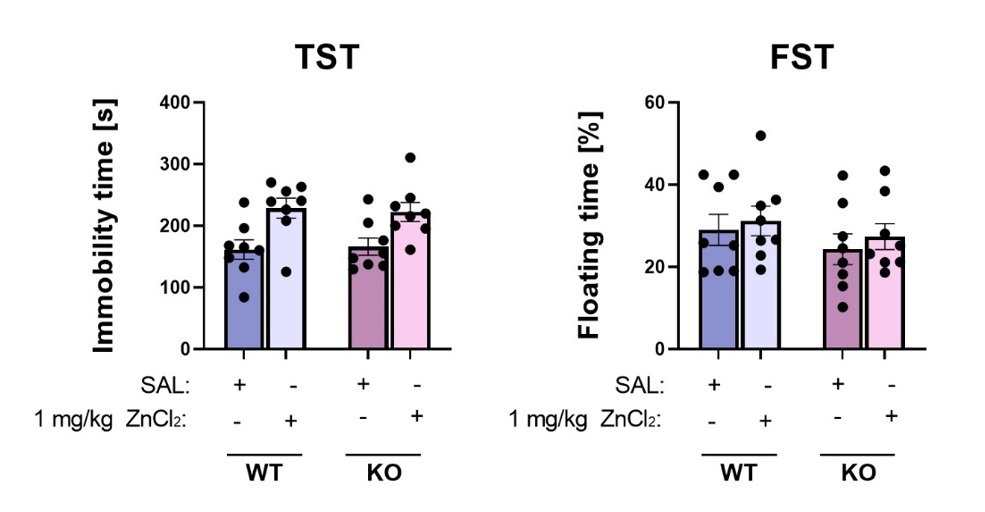

Supplement: Supplementary file 2 [file Image3.JPEG]

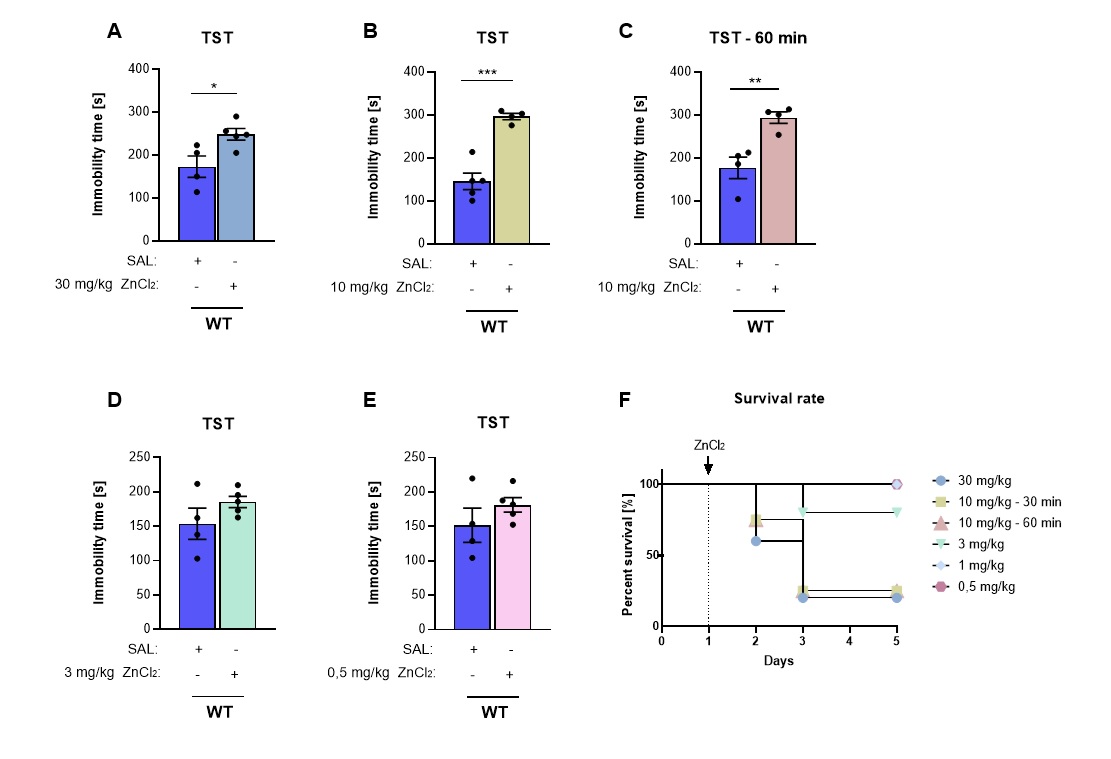

Supplement: Supplementary file 5 [file Image1.JPEG]

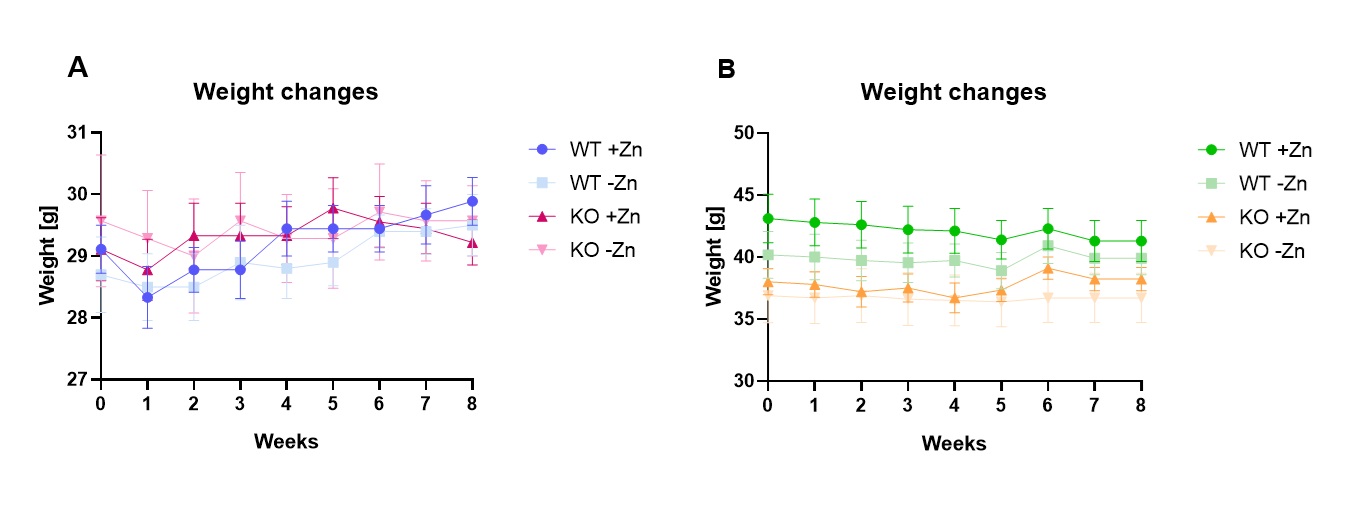

Supplement: Supplementary file 6 [file Image4.JPEG]

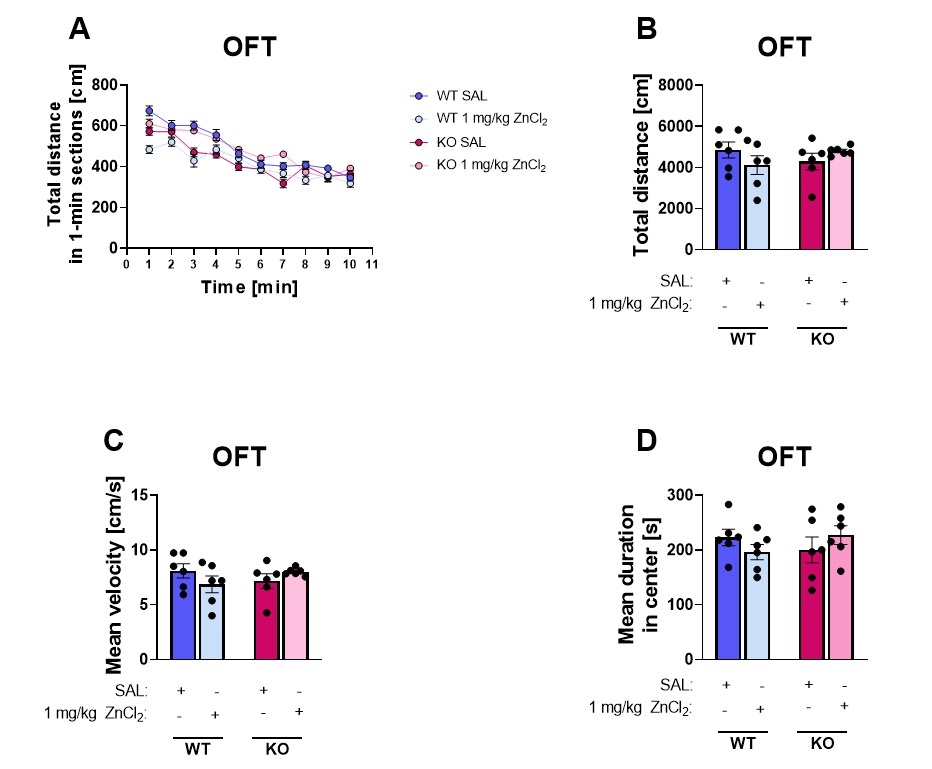

Supplement: Supplementary file 7 [file Image2.JPEG]

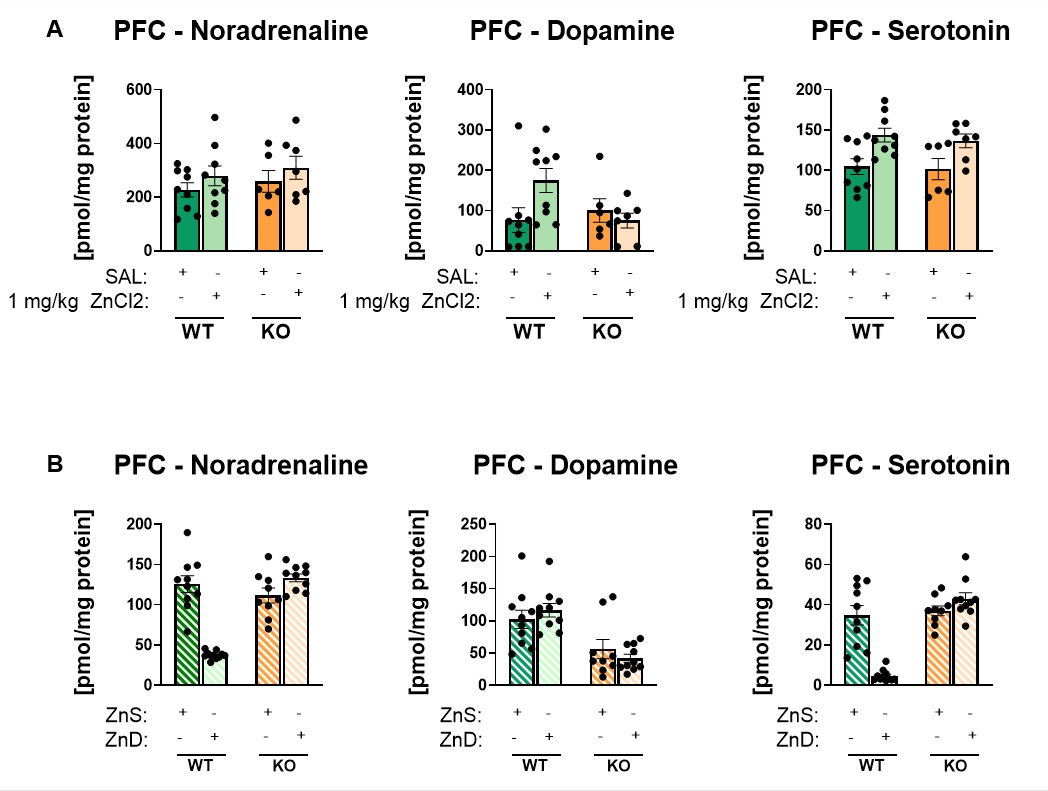

Supplement: Supplementary file 8 [file Image5.JPEG]

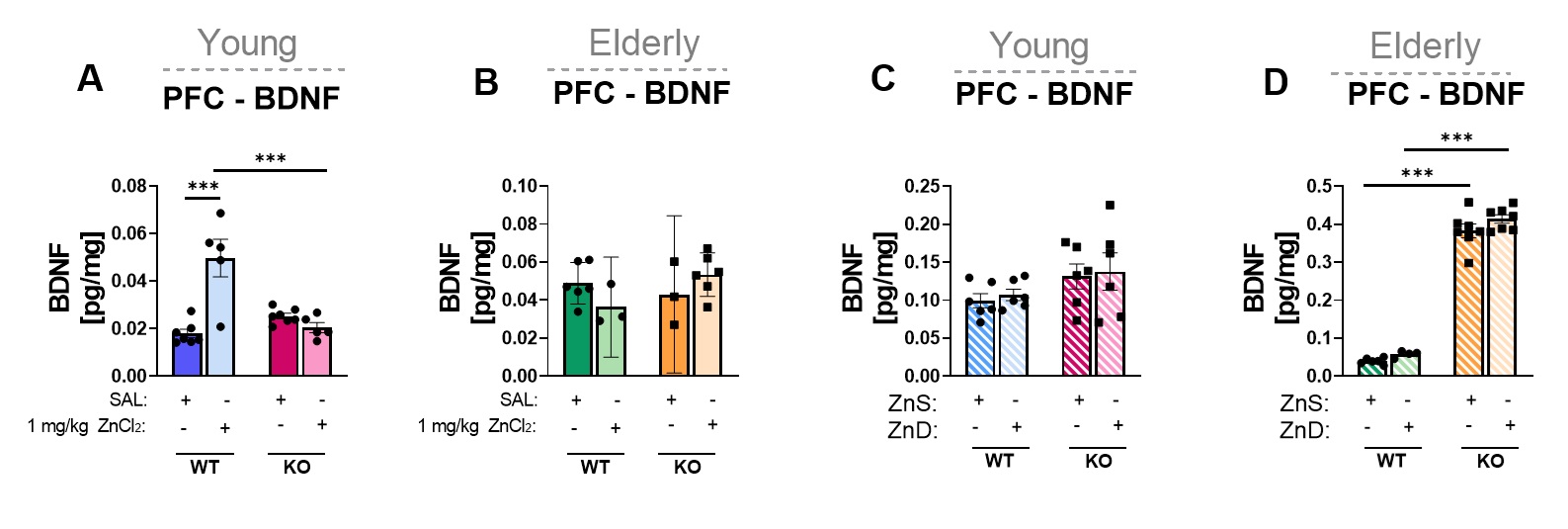

Supplement: Supplementary file 11 [file Image6.JPEG]
