# Supplementary material for: The antidepressant effect of short- and long-term zinc exposition is partly mediated by P2X7 receptors in male mice
Source: Front Pharmacol. 2023 Oct 16;14:1241406. doi: 10.3389/fphar.2023.1241406 (PMC10613712; doi:10.3389/fphar.2023.1241406)
Supplement: Supplementary file 4 [file Table1.DOCX]

Supplementary Material

**The antidepressant effect of short and long-term extracellular zinc is partly mediated by P2X7 receptors**

Bernadett Iring-Varga^1,2^, Mária Baranyi^1^, Flóra Gölöncsér^1^, Pál Tod^1^ and Beáta Sperlágh^1,2^ *

**Correspondence: Beáta Sperlágh**, [sperlagh@koki.hu](mailto:sperlagh@koki.hu)

**Supplementary Figure 1**. Preliminary TST experiments before the start of the study. All tests were performed on young, P2rx7 +/+ male mice. Different doses of ZnCl_2_ solution were injected into the animals (30 or 60 min before the start) to adjust the treatment: 30 (A) (n=4-5), 10 (B-C) (n=4-5), 3 (D) (n=4-5) and 0,5 (E) (n=4-6) mg/kg. Kaplan-Meier survival analysis performed on experimental groups receiving ZnCl2 solutions (F).

Data are expressed as mean ± S.E.M. Data were analyzed by three-way ANOWA followed by Tukey’s test. * p < 0,05; ** p < 0,01; *** p < 0,001.

TST: tail suspension test; WT: wild-type

**Supplementary Figure 2.** Zinc did not affect locomotor activity and anxiety in mice. (A) Distance travelled by time. (B) Total distance travelled (interaction: F[1, 20] = 2.938, p = 0.1020). (C) Velocity (interaction: F[1, 20] = 2.941, p = 0.1018). (D) Cumulative duration in central zone (interaction: F[1, 20] = 2.254, p = 0.1489). Values are presented as means ± SEM of n = 6 mice/group. Two-way ANOVA (B, C, D) followed by Tukey’s multiple comparison post hoc test.

OFT: open field test; WT: wild-type; KO: knock out

**Supplementary Figure 3.** Preliminary experiment performed on young female +/+ and -/- animals. According to the acute protocol, the mice received 1 mg/kg ZnCl_2_ solution or SAL i.p. 30 min. before the start of the TST and FST experiments (n=8/group).

Data are expressed as mean ± S.E.M. Data were analyzed by three-way ANOWA followed by Tukey’s test. * p < 0,05; ** p < 0,01; *** p < 0,001.

TST: tail suspension test; FST: forced swim test; WT: wild-type; KO: knock out

**Supplementary Figure 4.** Changes in the weight of young (A) (n=7-10) and elderly (B) (n=9-11) animals receiving chronic treatment as a result of different diets containing zinc.

WT: wild-type; KO: knock out; ZnS: zinc-supplemented diet; ZnD: zinc-deficient diet

**Supplementary Figure 5.** Monoamine content of Prefrontal Cortex was measured using HPLC technique. Noradrenaline, dopamine, and serotonin levels were plotted in P2rx7 +/+ and P2rx7 -/- elderly, acute (A) (n= 6-7) and chronic treated mice (B) (n= 9-11). Data are expressed as mean ± S.E.M. Data were analyzed by two-way ANOWA followed by Tukey’s test. * p < 0,05; ** p < 0,01; *** p < 0,001.

PFC: prefrontal cortex; WT: wild-type; KO: knock out; ZnS: zinc-supplemented diet; ZnD: zinc-deficient diet

**Supplementary Figure 6.** Examination of prefrontal cortex BDNF protein content by enzyme-linked immunosorbent assay (ELISA) (A-D). Wild type and knock out, young (n= 5-7) (A) and elderly (n=6) (B) mice were injected intraperitoneally with an acute treatment of saline or ZnCl_2_ (1 mg/kg). P2rx7 +/+ and -/- young (n= 3-6) (C) or elderly mice were fed Zn- controlled diet (n= 4-7) (D). Data are expressed as mean ± S.E.M. Data were analyzed by two-way ANOWA followed by Tukey’s test. * p < 0,05; ** p < 0,01; *** p < 0,001.

PFC: prefrontal cortex; WT: wild-type; KO: knock out; ZnS: zinc-supplemented diet; ZnD: zinc-deficient diet

| **Figure** | **Monoamine** | **Statistical Analysis** | **F-interaction value** | **p-value** |  | **Post hoc test** | **p-value** | **n** |  |
| --- | --- | --- | --- | --- | --- | --- | --- | --- | --- |
| **Fig. 2.A** |  | Two-way ANOVA | F interaction (1, 29) = 2,2780 | 0,142 | **n.s** | Tukey's post-hoc test |  | 8,7,8,10 |  |
|  |  |  | F treatment (1, 29) = 72,77 | <0,0001 |  | SAL:WT vs. SAL:KO | <0,0001 |  |  |
|  |  |  | F genotype (1, 29) = 43,84 | <0,0001 |  | SAL:WT vs. 1 mg/kg ZnCl_2_:WT | <0,0001 |  |  |
|  |  |  |  |  |  | SAL:WT vs. 1 mg/kg ZnCl_2_:KO | <0,0001 |  |  |
|  |  |  |  |  |  | SAL:KO vs. 1 mg/kg ZnCl_2_:WT | 0,7486 |  |  |
|  |  |  |  |  |  | SAL:KO vs. 1 mg/kg ZnCl_2_:KO | <0,0001 |  |  |
|  |  |  |  |  |  | 1 mg/kg ZnCl_2_:WT vs. 1 mg/kg ZnCl_2_:KO | 0,0063 |  |  |
|  |  | Two-way ANOVA | **F interaction (1, 29) = 5,409** | **0,0272** | ***** | Tukey's post-hoc test |  | 8,7,8,10 |  |
|  |  |  | F treatment (1, 29) = 54,78 | <0,0001 |  | **SAL:WT vs. SAL:KO** | **<0,0001** |  |  |
|  |  |  | F genotype (1, 29) = 42,37 | <0,0001 |  | **SAL:WT vs. 1 mg/kg ZnCl_2_:WT** | **<0,0001** |  |  |
|  |  |  |  |  |  | SAL:WT vs. 1 mg/kg ZnCl_2_:KO | <0,0001 |  |  |
|  |  |  |  |  |  | SAL:KO vs. 1 mg/kg ZnCl_2_:WT | 0,9319 |  |  |
|  |  |  |  |  |  | **SAL:KO vs. 1 mg/kg ZnCl_2_:KO** | **0,0061** |  |  |
|  |  |  |  |  |  | **1 mg/kg ZnCl_2_:WT vs. 1 mg/kg ZnCl_2_:KO** | **0,0391** |  |  |
| **Fig. 2.B** |  | Two-way ANOVA | F interaction (1, 30) = 0,45990 | 0,5029 | **n.s** | Tukey's post-hoc test |  | 10,9,6,9 |  |
|  |  |  | F treatment (1, 30) = 4,395 | 0,0446 |  | +Zn:WT vs. +Zn:KO | 0,9928 |  |  |
|  |  |  | F genotype (1, 30) = 0,09657 | 0,7581 |  | +Zn:WT vs. -Zn:WT | 0,1727 |  |  |
|  |  |  |  |  |  | +Zn:WT vs. -Zn:KO | 0,6411 |  |  |
|  |  |  |  |  |  | +Zn:KO vs. -Zn:WT | 0,2789 |  |  |
|  |  |  |  |  |  | +Zn:KO vs. -Zn:KO | 0,7833 |  |  |
|  |  |  |  |  |  | -Zn:WT vs. -Zn:KO | 0,9077 |  |  |
|  |  | Two-way ANOVA | F interaction (1, 31) = 0,6793 | 0,4161 | **n.s** | Tukey's post-hoc test |  | 10,9,6,9 |  |
|  |  |  | F treatment (1, 31) = 90,47 | <0,0001 |  | +Zn:WT vs. +Zn:KO | 0,1705 |  |  |
|  |  |  | F genotype (1, 31) = 4,414 | 0,0439 |  | +Zn:WT vs. -Zn:WT | <0,0001 |  |  |
|  |  |  |  |  |  | +Zn:WT vs. -Zn:KO | <0,0001 |  |  |
|  |  |  |  |  |  | +Zn:KO vs. -Zn:WT | <0,0001 |  |  |
|  |  |  |  |  |  | +Zn:KO vs. -Zn:KO | <0,0001 |  |  |
|  |  |  |  |  |  | -Zn:WT vs. -Zn:KO | 0,8134 |  |  |
| **Fig. 2.C** |  | Two-way ANOVA | **F interaction (1, 34) = 6,375** | **0,0164** | ***** | Tukey's post-hoc test |  | 10, 10, 10, 9 |  |
|  |  |  | F treatment (1, 34) = 15,82 | 0,0003 |  | SAL:WT vs. SAL:KO | 0,891 |  |  |
|  |  |  | F genotype (1, 34) = 2,297 | 0,1388 |  | **SAL:WT vs. 1 mg/kg ZnCl_2_:WT** | **0,0003** |  |  |
|  |  |  |  |  |  | SAL:WT vs. 1 mg/kg ZnCl_2_:KO | 0,3409 |  |  |
|  |  |  |  |  |  | SAL:KO vs. 1 mg/kg ZnCl_2_:WT | 0,0018 |  |  |
|  |  |  |  |  |  | SAL:KO vs. 1 mg/kg ZnCl_2_:KO | 0,7346 |  |  |
|  |  |  |  |  |  | **1 mg/kg ZnCl_2_:WT vs. 1 mg/kg ZnCl_2_:KO** | **0,0348** |  |  |
|  |  | Two-way ANOVA | F interaction (1, 34) = 0,2846 | 0,5972 | **n.s** | Tukey's post-hoc test |  | 10, 10, 10, 9 |  |
|  |  |  | F treatment (1, 34) = 21,81 | <0,0001 |  | SAL:WT vs. SAL:KO | 0,5571 |  |  |
|  |  |  | F genotype (1, 34) = 5,760 | 0,022 |  | SAL:WT vs. 1 mg/kg ZnCl_2_:WT | 0,0043 |  |  |
|  |  |  |  |  |  | SAL:WT vs. 1 mg/kg ZnCl_2_:KO | 0,4118 |  |  |
|  |  |  |  |  |  | SAL:KO vs. 1 mg/kg ZnCl_2_:WT | <0,0001 |  |  |
|  |  |  |  |  |  | SAL:KO vs. 1 mg/kg ZnCl_2_:KO | 0,0296 |  |  |
|  |  |  |  |  |  | 1 mg/kg ZnCl_2_:WT vs. 1 mg/kg ZnCl_2_:KO | 0,182 |  |  |
| **Fig. 2.D** |  | Two-way ANOVA | F interaction (1, 36) = 2,524 | 0,1209 | **n.s** | Tukey's post-hoc test |  | 10,9,6,9 |  |
|  |  |  | F treatment (1, 36) = 12,69 | 0,0011 |  | +Zn:WT vs. +Zn:KO | 0,0584 |  |  |
|  |  |  | F genotype (1, 36) = 5,542 | 0,0241 |  | +Zn:WT vs. -Zn:WT | 0,0039 |  |  |
|  |  |  |  |  |  | +Zn:WT vs. -Zn:KO | 0,001 |  |  |
|  |  |  |  |  |  | +Zn:KO vs. -Zn:WT | 0,9534 |  |  |
|  |  |  |  |  |  | +Zn:KO vs. -Zn:KO | 0,7002 |  |  |
|  |  |  |  |  |  | -Zn:WT vs. -Zn:KO | 0,9947 |  |  |
|  |  | Two-way ANOVA | **F interaction (1, 36) = 78,53** | **<0,0001** | ******* | Tukey's post-hoc test |  | 10,9,6,9 |  |
|  |  |  | F treatment (1, 36) = 21,81 | <0,0001 |  | **+Zn:WT vs. +Zn:KO** | **<0,0001** |  |  |
|  |  |  | F genotype (1, 36) = 5,760 | 0,022 |  | **+Zn:WT vs. -Zn:WT** | **<0,0001** |  |  |
|  |  |  |  |  |  | +Zn:WT vs. -Zn:KO | <0,0001 |  |  |
|  |  |  |  |  |  | +Zn:KO vs. -Zn:WT | 0,3792 |  |  |
|  |  |  |  |  |  | +Zn:KO vs. -Zn:KO | 0,4181 |  |  |
|  |  |  |  |  |  | **-Zn:WT vs. -Zn:KO** | **0,0112** |  |  |
| **Fig. 3.A** |  | Two-way ANOVA | F interaction (1, 36) = 3,154 | 0,0874 | **n.s** | Tukey's post-hoc test |  | 8, 8, 6, 8 |  |
|  |  |  | F treatment (1, 36) = 43,73 | <0,0001 |  | SAL:WT vs. SAL:KO | 0,9298 |  |  |
|  |  |  | F _genotype_ (1, 36) = 18,6 | 0,0001 |  | SAL:WT vs. 1 mg/kg ZnCl_2_:WT | <0,0001 |  |  |
|  |  |  |  |  |  | SAL:WT vs. 1 mg/kg ZnCl_2_:KO | 0,0179 |  |  |
|  |  |  |  |  |  | SAL:KO vs. 1 mg/kg ZnCl_2_:WT | <0,0001 |  |  |
|  |  |  |  |  |  | SAL:KO vs. 1 mg/kg ZnCl_2_:KO | 0,0074 |  |  |
|  |  |  |  |  |  | 1 mg/kg ZnCl_2_:WT vs. 1 mg/kg ZnCl_2_:KO | 0,0152 |  |  |
| **Fig. 3.B** |  | Two-way ANOVA | F interaction (1, 18) = 0,03277 | 0,8584 | **n.s** | Tukey's post-hoc test |  | 6, 6, 6, 4 |  |
|  |  |  | F treatment (1, 18) = 3,172 | 0,0918 |  | +Zn:WT vs. +Zn:KO | 0,962 |  |  |
|  |  |  | F _genotype_ (1, 18) =0,6795 | 0,4206 |  | +Zn:WT vs. -Zn:WT | 0,6346 |  |  |
|  |  |  |  |  |  | +Zn:WT vs. -Zn:KO | 0,3294 |  |  |
|  |  |  |  |  |  | +Zn:KO vs. -Zn:WT | 0,8888 |  |  |
|  |  |  |  |  |  | +Zn:KO vs. -Zn:KO | 0,5649 |  |  |
|  |  |  |  |  |  | -Zn:WT vs. -Zn:KO | 0,9054 |  |  |
| **Fig. 3.C** |  | Two-way ANOVA | **F interaction (1, 26) = 16,58** | **0,0004** | ******* | Tukey's post-hoc test |  | 7, 8, 7, 7 |  |
|  |  |  | F treatment (1, 26) = 149,8 | <0,0001 |  | **SAL:WT vs. SAL:KO** | **0,0137** |  |  |
|  |  |  | F _genotype_ (1, 26) =0,3667 | 0,5495 |  | **SAL:WT vs. 1 mg/kg ZnCl_2_:WT** | **<0,0001** |  |  |
|  |  |  |  |  |  | SAL:WT vs. 1 mg/kg ZnCl_2_:KO | <0,0001 |  |  |
|  |  |  |  |  |  | SAL:KO vs. 1 mg/kg ZnCl_2_:WT | <0,0001 |  |  |
|  |  |  |  |  |  | **SAL:KO vs. 1 mg/kg ZnCl_2_:KO** | **<0,0001** |  |  |
|  |  |  |  |  |  | 1 mg/kg ZnCl_2_:WT vs. 1 mg/kg ZnCl_2_:KO | 0,0923 |  |  |
| **Fig. 3.D** |  | Two-way ANOVA | F interaction (1, 29) = 1,736 | 0,1979 | **n.s** | Tukey's post-hoc test |  | 8, 9, 8, 8 |  |
|  |  |  | F treatment (1, 29) = 3,189 | 0,0846 |  | +Zn:WT vs. +Zn:KO | 0,9872 |  |  |
|  |  |  | F _genotype_ (1, 29) =0,7106 | 0,4061 |  | +Zn:WT vs. -Zn:WT | 0,1399 |  |  |
|  |  |  |  |  |  | +Zn:WT vs. -Zn:KO | 0,9121 |  |  |
|  |  |  |  |  |  | +Zn:KO vs. -Zn:WT | 0,256 |  |  |
|  |  |  |  |  |  | +Zn:KO vs. -Zn:KO | 0,9877 |  |  |
|  |  |  |  |  |  | -Zn:WT vs. -Zn:KO | 0,422 |  |  |
| **Fig. 4.A** | *Noradrenaline* | Two-way ANOVA | F interaction (1, 21) = 5,606 | 0,0276 | ***** | Tukey's post-hoc test |  | 9, 7, 5, 5, |  |
|  |  |  | F treatment (1, 21) = 28,06 | <0,0001 |  | **SAL:WT vs. SAL:KO** | **0,025** |  |  |
|  |  |  | F _genotype_ (1, 21) =3,144 | 0,0907 |  | SAL:WT vs. 1 mg/kg ZnCl_2_:WT | 0,1002 |  |  |
|  |  |  |  |  |  | SAL:WT vs. 1 mg/kg ZnCl_2_:KO | 0,0961 |  |  |
|  |  |  |  |  |  | SAL:KO vs. 1 mg/kg ZnCl_2_:WT | 0,0003 |  |  |
|  |  |  |  |  |  | **SAL:KO vs. 1 mg/kg ZnCl_2_:KO** | **0,0005** |  |  |
|  |  |  |  |  |  | **1 mg/kg ZnCl_2_:WT vs. 1 mg/kg ZnCl_2_:KO** | **0,9781** |  |  |
|  | *Dopamine* | Two-way ANOVA | F interaction (1, 21) = 6,451 | 0,0191 | ***** | Tukey's post-hoc test |  | 9, 7, 5, 5, |  |
|  |  |  | F treatment (1, 21) = 9,634 | 0,0054 |  | SAL:WT vs. SAL:KO | 0,9177 |  |  |
|  |  |  | F _genotype_ (1, 21) =2,850 | 0,1061 |  | SAL:WT vs. 1 mg/kg ZnCl_2_:WT | 0,9649 |  |  |
|  |  |  |  |  |  | SAL:WT vs. 1 mg/kg ZnCl_2_:KO | 0,015 |  |  |
|  |  |  |  |  |  | SAL:KO vs. 1 mg/kg ZnCl_2_:WT | 0,7431 |  |  |
|  |  |  |  |  |  | **SAL:KO vs. 1 mg/kg ZnCl_2_:KO** | **0,0099** |  |  |
|  |  |  |  |  |  | **1 mg/kg ZnCl_2_:WT vs. 1 mg/kg ZnCl_2_:KO** | **0,0458** |  |  |
|  | *Serotonin* | Two-way ANOVA | F interaction (1, 22) = 0,03745 | 0,8483 | **n.s** | Tukey's post-hoc test |  | 9, 8, 5, 5, |  |
|  |  |  | F treatment (1, 22) = 12,81 | 0,0017 |  | SAL:WT vs. SAL:KO | 0,9085 |  |  |
|  |  |  | F _genotype_ (1, 22) = 1,191 | 0,2869 |  | SAL:WT vs. 1 mg/kg ZnCl_2_:WT | 0,0193 |  |  |
|  |  |  |  |  |  | SAL:WT vs. 1 mg/kg ZnCl_2_:KO | 0,3401 |  |  |
|  |  |  |  |  |  | SAL:KO vs. 1 mg/kg ZnCl_2_:WT | 0,0129 |  |  |
|  |  |  |  |  |  | SAL:KO vs. 1 mg/kg ZnCl_2_:KO | 0,0097 |  |  |
|  |  |  |  |  |  | 1 mg/kg ZnCl_2_:WT vs. 1 mg/kg ZnCl_2_:KO | 0,8206 |  |  |
| **Fig. 4.B** | *Noradrenaline* | Two-way ANOVA | F interaction (1, 22) = 0,4623 | 0,5036 | **n.s** | Tukey's post-hoc test |  | 6, 7, 6, 7 |  |
|  |  |  | F treatment (1, 22) = 1,570 | 0,2234 |  | +Zn:WT vs. +Zn:KO | 0,9969 |  |  |
|  |  |  | F _genotype_ (1, 22) = 0,1449 | 0,7071 |  | +Zn:WT vs. -Zn:WT | 0,5324 |  |  |
|  |  |  |  |  |  | +Zn:WT vs. -Zn:KO | 0,9256 |  |  |
|  |  |  |  |  |  | +Zn:KO vs. -Zn:WT | 0,6604 |  |  |
|  |  |  |  |  |  | +Zn:KO vs. -Zn:KO | 0,977 |  |  |
|  |  |  |  |  |  |  |  |  |  |
|  |  |  |  |  |  | -Zn:WT vs. -Zn:KO | 0,8624 |  |  |
|  | *Dopamine* | Two-way ANOVA | F interaction (1, 22) = 0,3855 | 0,541 | **n.s** | Tukey's post-hoc test |  | 6, 7, 6, 7 |  |
|  |  |  | F treatment (1, 22) = 1,988 | 0,1726 |  | +Zn:WT vs. +Zn:KO | 0,7677 |  |  |
|  |  |  | F _genotype_ (1, 22) = 0,6441 | 0,4308 |  | +Zn:WT vs. -Zn:WT | 0,9434 |  |  |
|  |  |  |  |  |  | +Zn:WT vs. -Zn:KO | 0,9728 |  |  |
|  |  |  |  |  |  | +Zn:KO vs. -Zn:WT | 0,4184 |  |  |
|  |  |  |  |  |  | +Zn:KO vs. -Zn:KO | 0,4914 |  |  |
|  |  |  |  |  |  | -Zn:WT vs. -Zn:KO | 0,9991 |  |  |
|  | *Serotonin* | Two-way ANOVA | **F interaction (1, 23) = 21,87** | **0,0001** | ******* | Tukey's post-hoc test |  | 7, 7, 6, 7 |  |
|  |  |  | F treatment (1, 23) = 3,423 | 0,0772 |  | **+Zn:WT vs. +Zn:KO** | **0,0002** |  |  |
|  |  |  | F _genotype_ (1, 23) = 7,859 | 0,0101 |  | **+Zn:WT vs. -Zn:WT** | **0,0005** |  |  |
|  |  |  |  |  |  | +Zn:WT vs. -Zn:KO | 0,0135 |  |  |
|  |  |  |  |  |  | +Zn:KO vs. -Zn:WT | 0,9106 |  |  |
|  |  |  |  |  |  | +Zn:KO vs. -Zn:KO | 0,2321 |  |  |
|  |  |  |  |  |  | -Zn:WT vs. -Zn:KO | 0,5409 |  |  |
| **Fig. 4.C** | *Noradrenaline* | Two-way ANOVA | F interaction (1, 27) = 0,005913 | 0,9393 | **n.s** | Tukey's post-hoc test |  | 9, 9, 6, 7 |  |
|  |  |  | F treatment (1, 27) = 0,6902 | 0,4134 |  | SAL:WT vs. SAL:KO | 0,997 |  |  |
|  |  |  | F _genotype_ (1, 27) = 0,1353 | 0,7159 |  | SAL:WT vs. 1 mg/kg ZnCl_2_:WT | 0,9363 |  |  |
|  |  |  |  |  |  | SAL:WT vs. 1 mg/kg ZnCl_2_:KO | 0,8216 |  |  |
|  |  |  |  |  |  | SAL:KO vs. 1 mg/kg ZnCl_2_:WT | 0,9884 |  |  |
|  |  |  |  |  |  | SAL:KO vs. 1 mg/kg ZnCl_2_:KO | 0,9328 |  |  |
|  |  |  |  |  |  | 1 mg/kg ZnCl_2_:WT vs. 1 mg/kg ZnCl_2_:KO | 0,9882 |  |  |
|  | *Dopamine* | Two-way ANOVA | F interaction (1, 27) = 0,2542 | 0,6182 | **n.s** | Tukey's post-hoc test |  | 9, 9, 6, 7 |  |
|  |  |  | F treatment (1, 27) = 1,203 | 0,2823 |  | SAL:WT vs. SAL:KO | 0,9663 |  |  |
|  |  |  | F _genotype_ (1, 27) = 0,02760 | 0,8693 |  | SAL:WT vs. 1 mg/kg ZnCl_2_:WT | 0,6086 |  |  |
|  |  |  |  |  |  | SAL:WT vs. 1 mg/kg ZnCl_2_:KO | 0,7977 |  |  |
|  |  |  |  |  |  | SAL:KO vs. 1 mg/kg ZnCl_2_:WT | 0,9167 |  |  |
|  |  |  |  |  |  | SAL:KO vs. 1 mg/kg ZnCl_2_:KO | 0,9797 |  |  |
|  |  |  |  |  |  | 1 mg/kg ZnCl_2_:WT vs. 1 mg/kg ZnCl_2_:KO | 0,9947 |  |  |
|  | *Serotonin* | Two-way ANOVA | F interaction (1, 27) = 0,7987 | 0,3794 | **n.s** | Tukey's post-hoc test |  | 9, 9, 6, 7 |  |
|  |  |  | F treatment (1, 27) = 8,431 | 0,0073 |  | SAL:WT vs. SAL:KO | 0,6214 |  |  |
|  |  |  | F _genotype_ (1, 27) =0,7502 | 0,394 |  | SAL:WT vs. 1 mg/kg ZnCl_2_:WT | 0,032 |  |  |
|  |  |  |  |  |  | SAL:WT vs. 1 mg/kg ZnCl_2_:KO | 0,051 |  |  |
|  |  |  |  |  |  | SAL:KO vs. 1 mg/kg ZnCl_2_:WT | 0,5045 |  |  |
|  |  |  |  |  |  | SAL:KO vs. 1 mg/kg ZnCl_2_:KO | 0,5603 |  |  |
|  |  |  |  |  |  | 1 mg/kg ZnCl_2_:WT vs. 1 mg/kg ZnCl_2_:KO | >0,9999 |  |  |
| **Fig. 4.D** | *Noradrenaline* | Two-way ANOVA | F interaction (1, 36) = 2,628 | 0,1137 | **n.s** | Tukey's post-hoc test |  | 10, 11, 9, 10 |  |
|  |  |  | F treatment (1, 36) = 25,19 | <0,0001 |  | -Zn:WT vs. -Zn:KO | 0,0014 |  |  |
|  |  |  | F _genotype_ (1, 36) =51,88 | <0,0001 |  | -Zn:WT vs. +Zn:WT | 0,0001 |  |  |
|  |  |  |  |  |  | -Zn:WT vs. +Zn:KO | 0,4246 |  |  |
|  |  |  |  |  |  | -Zn:KO vs. +Zn:WT | <0,0001 |  |  |
|  |  |  |  |  |  | -Zn:KO vs. +Zn:KO | 0,1069 |  |  |
|  |  |  |  |  |  | +Zn:WT vs. +Zn:KO | <0,0001 |  |  |
|  | *Dopamine* | Two-way ANOVA | F interaction (1, 36) = 1,475 | 0,2325 | **n.s** | Tukey's post-hoc test |  | 10, 11, 9, 10 |  |
|  |  |  | F treatment (1, 36) = 13,27 | 0,0008 |  | -Zn:WT vs. -Zn:KO | 0,5155 |  |  |
|  |  |  | F _genotype_ (1, 36) =9,771 | 0,0035 |  | -Zn:WT vs. +Zn:WT | 0,0062 |  |  |
|  |  |  |  |  |  | -Zn:WT vs. +Zn:KO | 0,9831 |  |  |
|  |  |  |  |  |  | -Zn:KO vs. +Zn:WT | 0,0002 |  |  |
|  |  |  |  |  |  | -Zn:KO vs. +Zn:KO | 0,351 |  |  |
|  |  |  |  |  |  | +Zn:WT vs. +Zn:KO | 0,0244 |  |  |
|  | *Serotonin* | Two-way ANOVA | F interaction (1, 36) = 0,5137 | 0,4782 | **n.s** | Tukey's post-hoc test |  | 10, 11, 9, 10 |  |
|  |  |  | F treatment (1, 36) = 24,25 | <0,0001 |  | -Zn:WT vs. -Zn:KO | <0,0001 |  |  |
|  |  |  | F _genotype_ (1, 36) =41,18 | <0,0001 |  | -Zn:WT vs. +Zn:WT | 0,0211 |  |  |
|  |  |  |  |  |  | -Zn:WT vs. +Zn:KO | 0,7199 |  |  |
|  |  |  |  |  |  | -Zn:KO vs. +Zn:WT | <0,0001 |  |  |
|  |  |  |  |  |  | -Zn:KO vs. +Zn:KO | 0,0022 |  |  |
|  |  |  |  |  |  | +Zn:WT vs. +Zn:KO | 0,002 |  |  |
| **Fig. 5.A** |  | Two-way ANOVA | F interaction (1, 25) = 0,8278 | 0,3716 | **n.s** | Tukey's post-hoc test |  | 11, 7, 7, 5 |  |
|  |  |  | F treatment (1, 25) = 0,03456 | 0,854 |  | SAL:WT vs. SAL:KO | 0,0105 |  |  |
|  |  |  | F _genotype_ (1, 25) = 12,33 | 0,0017 |  | SAL:WT vs. ZnCl2:WT | 0,8297 |  |  |
|  |  |  |  |  |  | SAL:WT vs. ZnCl2:KO | 0,0713 |  |  |
|  |  |  |  |  |  | SAL:KO vs. ZnCl2:WT | 0,1071 |  |  |
|  |  |  |  |  |  | SAL:KO vs. ZnCl2:KO | 0,9643 |  |  |
|  |  |  |  |  |  | ZnCl2:WT vs. ZnCl2:KO | 0,3448 |  |  |
| **Fig. 5.B** |  | Two-way ANOVA | F interaction (1, 20) = 3,856 | 0,0636 | **n.s** | Tukey's post-hoc test |  | 6, 6, 6, 6 |  |
|  |  |  | F treatment (1, 20) = 0,0041 | 0,9498 |  | +Zn:WT vs. +Zn:KO | 0,0159 |  |  |
|  |  |  | F _genotype_ (1, 20) =7,597 | 0,0122 |  | +Zn:WT vs. -Zn:WT | 0,4941 |  |  |
|  |  |  |  |  |  | +Zn:WT vs. -Zn:KO | 0,2233 |  |  |
|  |  |  |  |  |  | +Zn:KO vs. -Zn:WT | 0,258 |  |  |
|  |  |  |  |  |  | +Zn:KO vs. -Zn:KO | 0,5475 |  |  |
|  |  |  |  |  |  | -Zn:WT vs. -Zn:KO | 0,9426 |  |  |
| **Fig. 5.C** |  | Two-way ANOVA | F interaction (1, 26) = 2,802 | 0,1061 | **n.s** | Tukey's post-hoc test |  | 11, 7, 6, 6 |  |
|  |  |  | F treatment (1, 26) = 0,0508 | 0,8234 |  | SAL:WT vs. SAL:KO | 0,0966 |  |  |
|  |  |  | F _genotype_ (1, 26) =2,08 | 0,1608 |  | SAL:WT vs. ZnCl2:WT | 0,5051 |  |  |
|  |  |  |  |  |  | SAL:WT vs. ZnCl2:KO | 0,6089 |  |  |
|  |  |  |  |  |  | SAL:KO vs. ZnCl2:WT | 0,8424 |  |  |
|  |  |  |  |  |  | SAL:KO vs. ZnCl2:KO | 0,7618 |  |  |
|  |  |  |  |  |  | ZnCl2:WT vs. ZnCl2:KO | 0,9988 |  |  |
| **Fig. 5.D** |  | Two-way ANOVA | F interaction (1, 35) = 1,487 | 0,2308 | **n.s** | Tukey's post-hoc test |  | 10, 11, 9, 9 |  |
|  |  |  | F treatment (1, 35) = 10,65 | 0,0025 |  | +Zn:WT vs. +Zn:KO | <0,0001 |  |  |
|  |  |  | F _genotype_ (1, 35) =71,22 | <0,0001 |  | +Zn:WT vs. -Zn:WT | 0,0115 |  |  |
|  |  |  |  |  |  | +Zn:WT vs. -Zn:KO | 0,0049 |  |  |
|  |  |  |  |  |  | +Zn:KO vs. -Zn:WT | <0,0001 |  |  |
|  |  |  |  |  |  | +Zn:KO vs. -Zn:KO | 0,5117 |  |  |
|  |  |  |  |  |  | -Zn:WT vs. -Zn:KO | <0,0001 |  |  |
| **Figure** |  | **Statistical Analysis** | **One- or two-tailed P value?** | **p-value** |  |  | | **n** |  |
| **Suppl fig.1.A** |  | Unpaired t-test | Two-tailed | **0,026** | ***** |  | | 4, 5 |  |
|  |  |  |  |  |  |  |  |  |  |
| **Suppl fig.1.B** |  | Unpaired t-test | Two-tailed | **0,0003** | ******* |  | | 5, 4 |  |
|  |  |  |  |  |  |  |  |  |  |
| **Suppl fig.1.C** |  | Unpaired t-test | Two-tailed | **0,0062** | ****** |  | | 4, 4 |  |
|  |  |  |  |  |  |  |  |  |  |
| **Suppl fig.1.D** |  | Unpaired t-test | Two-tailed | 0,2712 | **n.s** |  | | 4, 5 |  |
|  |  |  |  |  |  |  |  |  |  |
| **Suppl fig.1.E** |  | Unpaired t-test | Two-tailed | 0,1953 | **n.s** |  | | 4, 5 |  |
|  |  |  |  |  |  |  |  |  |  |
| **Figure** | **Experiment** | **Statistical Analysis** | **F-interaction value** | **p-value** |  | **Post hoc test** | **p-value** | **n** |  |
| **Suppl. Fig. 2. B** |  | Two-way ANOVA | F interaction (1, 20) = 2,938 | 0,102 | **n.s** | Tukey's post-hoc test |  | 6, 6, 6, 6 |  |
|  |  |  | F treatment (1, 20) = 0,1017 | 0,7531 |  | SAL:WT vs. SAL:KO | 0,6982 |  |  |
|  |  |  | F _genotype_ (1, 20) = 0,02873 | 0,8671 |  | SAL:WT vs. ZnCl2:WT | 0,4918 |  |  |
|  |  |  |  |  |  | SAL:WT vs. ZnCl2:KO | 0,9996 |  |  |
|  |  |  |  |  |  | SAL:KO vs. ZnCl2:WT | 0,9854 |  |  |
|  |  |  |  |  |  | SAL:KO vs. ZnCl2:KO | 0,7586 |  |  |
|  |  |  |  |  |  | ZnCl2:WT vs. ZnCl2:KO | 0,5543 |  |  |
| **Suppl. Fig. 2. C** |  | Two-way ANOVA | F interaction (1, 20) = 2,941 | 0,1018 | **n.s** | Tukey's post-hoc test |  | 6, 6, 6, 6 |  |
|  |  |  | F treatment (1, 20) = 0,1025 | 0,7522 |  | SAL:WT vs. SAL:KO | 0,6968 |  |  |
|  |  |  | F _genotype_ (1, 20) =0,02789 | 0,869 |  | SAL:WT vs. ZnCl2:WT | 0,4909 |  |  |
|  |  |  |  |  |  | SAL:WT vs. ZnCl2:KO | 0,9995 |  |  |
|  |  |  |  |  |  | SAL:KO vs. ZnCl2:WT | 0,9855 |  |  |
|  |  |  |  |  |  | SAL:KO vs. ZnCl2:KO | 0,7588 |  |  |
|  |  |  |  |  |  | ZnCl2:WT vs. ZnCl2:KO | 0,555 |  |  |
| **Suppl. Fig. 2. D** |  | Two-way ANOVA | F interaction (1, 20) = 2,254 | 0,1489 | **n.s** | Tukey's post-hoc test |  | 6, 6, 6, 6 |  |
|  |  |  | F treatment (1, 20) = 0,0002328 | 0,988 |  | SAL:WT vs. SAL:KO | 0,8094 |  |  |
|  |  |  | F _genotype_ (1, 20) =0,05810 | 0,812 |  | SAL:WT vs. ZnCl2:WT | 0,7222 |  |  |
|  |  |  |  |  |  | SAL:WT vs. ZnCl2:KO | 0,9978 |  |  |
|  |  |  |  |  |  | SAL:KO vs. ZnCl2:WT | 0,9985 |  |  |
|  |  |  |  |  |  | SAL:KO vs. ZnCl2:KO | 0,7097 |  |  |
|  |  |  |  |  |  | ZnCl2:WT vs. ZnCl2:KO | 0,6145 |  |  |
| **Suppl fig. 3** | TST | Two-way ANOVA | F interaction (1, 28) = 0,1220 | 0,7294 | **n.s** | Tukey's post-hoc test |  | 8, 8, 8, 8 |  |
|  |  |  | F treatment (1, 28) = 15,94 | 0,0004 |  | SAL:WT vs. SAL:KO | 0,9964 |  |  |
|  |  |  | F _genotype_ (1, 28) = 0,002125 | 0,9636 |  | SAL:WT vs. 1 mg/kg ZnCl_2_:WT | 0,023 |  |  |
|  |  |  |  |  |  | SAL:WT vs. 1 mg/kg ZnCl_2_:KO | 0,0438 |  |  |
|  |  |  |  |  |  | SAL:KO vs. 1 mg/kg ZnCl_2_:WT | 0,0378 |  |  |
|  |  |  |  |  |  | SAL:KO vs. 1 mg/kg ZnCl_2_:KO | 0,0698 |  |  |
|  |  |  |  |  |  | 1 mg/kg ZnCl_2_:WT vs. 1 mg/kg ZnCl_2_:KO | 0,9922 |  |  |
|  | FST | Two-way ANOVA | F interaction (1, 28) = 0,01623 | 0,8995 | **n.s** | Tukey's post-hoc test |  | 8, 8, 8, 8 |  |
|  |  |  | F treatment (1, 28) = 0,5411 | 0,4681 |  | SAL:WT vs. SAL:KO | 0,7879 |  |  |
|  |  |  | F _genotype_ (1, 28) =1,419 | 0,2436 |  | SAL:WT vs. 1 mg/kg ZnCl_2_:WT | 0,9728 |  |  |
|  |  |  |  |  |  | SAL:WT vs. 1 mg/kg ZnCl_2_:KO | 0,9882 |  |  |
|  |  |  |  |  |  | SAL:KO vs. 1 mg/kg ZnCl_2_:WT | 0,5325 |  |  |
|  |  |  |  |  |  | SAL:KO vs. 1 mg/kg ZnCl_2_:KO | 0,928 |  |  |
|  |  |  |  |  |  | 1 mg/kg ZnCl_2_:WT vs. 1 mg/kg ZnCl_2_:KO | 0,875 |  |  |
| **Suppl fig. 5.A** | *Noradrenaline* | Two-way ANOVA | F interaction (1, 27) = 0,0005260 | 0,9819 | **n.s** | Tukey's post-hoc test |  | 9, 9, 6, 7 |  |
|  |  |  | F treatment (1, 27) = 1,963 | 0,1726 |  | SAL:WT vs. SAL:KO | 0,9326 |  |  |
|  |  |  | F _genotype_ (1, 27) =0,7018 | 0,4095 |  | SAL:WT vs. 1 mg/kg ZnCl2:WT | 0,6917 |  |  |
|  |  |  |  |  |  | SAL:WT vs. 1 mg/kg ZnCl2:KO | 0,3849 |  |  |
|  |  |  |  |  |  | SAL:KO vs. 1 mg/kg ZnCl2:WT | 0,9795 |  |  |
|  |  |  |  |  |  | SAL:KO vs. 1 mg/kg ZnCl2:KO | 0,8032 |  |  |
|  |  |  |  |  |  | 1 mg/kg ZnCl2:WT vs. 1 mg/kg ZnCl2:KO | 0,9344 |  |  |
|  | *Dopamine* | Two-way ANOVA | **F interaction (1, 27) = 4,464** | **0,044** | ***** | Tukey's post-hoc test |  | 9, 9, 6, 7 |  |
|  |  |  | F treatment (1, 27) = 1,592 | 0,2179 |  | SAL:WT vs. SAL:KO | 0,9414 |  |  |
|  |  |  | F _genotype_ (1, 27) =1,678 | 0,2062 |  | SAL:WT vs. 1 mg/kg ZnCl2:WT | 0,0657 |  |  |
|  |  |  |  |  |  | SAL:WT vs. 1 mg/kg ZnCl2:KO | >0,9999 |  |  |
|  |  |  |  |  |  | SAL:KO vs. 1 mg/kg ZnCl2:WT | 0,3098 |  |  |
|  |  |  |  |  |  | SAL:KO vs. 1 mg/kg ZnCl2:KO | 0,9436 |  |  |
|  |  |  |  |  |  | 1 mg/kg ZnCl2:WT vs. 1 mg/kg ZnCl2:KO | 0,0886 |  |  |
|  | *Serotonin* | Two-way ANOVA | F interaction (1, 27) = 0,03403 | 0,855 | **n.s** | Tukey's post-hoc test |  | 9, 9, 6, 7 |  |
|  |  |  | F treatment (1, 27) = 13,63 | 0,001 |  | SAL:WT vs. SAL:KO | 0,9961 |  |  |
|  |  |  | F _genotype_ (1, 27) =0,2527 | 0,6192 |  | SAL:WT vs. 1 mg/kg ZnCl2:WT | 0,0278 |  |  |
|  |  |  |  |  |  | SAL:WT vs. 1 mg/kg ZnCl2:KO | 0,1214 |  |  |
|  |  |  |  |  |  | SAL:KO vs. 1 mg/kg ZnCl2:WT | 0,0346 |  |  |
|  |  |  |  |  |  | SAL:KO vs. 1 mg/kg ZnCl2:KO | 0,1234 |  |  |
|  |  |  |  |  |  | 1 mg/kg ZnCl2:WT vs. 1 mg/kg ZnCl2:KO | 0,959 |  |  |
| **Suppl fig. 5.B** | *Noradrenaline* | Two-way ANOVA | **F interaction (1, 36) = 58,75** | **<0,0001** | *** | Tukey's post-hoc test |  | 10, 11, 9, 10 |  |
|  |  |  | F treatment (1, 36) = 21,41 | <0,0001 |  | **-Zn:WT vs. -Zn:KO** | **<0,0001** |  |  |
|  |  |  | F _genotype_ (1, 36) =32,52 | <0,0001 |  | **-Zn:WT vs. +Zn:WT** | **<0,0001** |  |  |
|  |  |  |  |  |  | -Zn:WT vs. +Zn:KO | <0,0001 |  |  |
|  |  |  |  |  |  | -Zn:KO vs. +Zn:WT | 0,8708 |  |  |
|  |  |  |  |  |  | -Zn:KO vs. +Zn:KO | 0,1738 |  |  |
|  |  |  |  |  |  | +Zn:WT vs. +Zn:KO | 0,5356 |  |  |
|  | *Dopamine* | Two-way ANOVA | F interaction (1, 35) = 1,424 | 0,2408 | **n.s** | Tukey's post-hoc test |  | 10, 10, 9, 10 |  |
|  |  |  | F treatment (1, 35) = 1,061e-005 | 0,9974 |  | -Zn:WT vs. -Zn:KO | 0,0004 |  |  |
|  |  |  | F _genotype_ (1, 35) =25,86 | <0,0001 |  | -Zn:WT vs. +Zn:WT | 0,8287 |  |  |
|  |  |  |  |  |  | -Zn:WT vs. +Zn:KO | 0,006 |  |  |
|  |  |  |  |  |  | -Zn:KO vs. +Zn:WT | 0,0045 |  |  |
|  |  |  |  |  |  | -Zn:KO vs. +Zn:KO | 0,8376 |  |  |
|  |  |  |  |  |  | +Zn:WT vs. +Zn:KO | 0,0479 |  |  |
|  | *Serotonin* | Two-way ANOVA | **F interaction (1, 36) = 34,44** | **<0,0001** | *** | Tukey's post-hoc test |  | 10, 11, 9, 10 |  |
|  |  |  | F treatment (1, 36) = 15,48 | 0,0004 |  | **-Zn:WT vs. -Zn:KO** | **<0,0001** |  |  |
|  |  |  | F _genotype_ (1, 36) =43,40 | <0,0001 |  | **-Zn:WT vs. +Zn:WT** | **<0,0001** |  |  |
|  |  |  |  |  |  | -Zn:WT vs. +Zn:KO | <0,0001 |  |  |
|  |  |  |  |  |  | -Zn:KO vs. +Zn:WT | 0,254 |  |  |
|  |  |  |  |  |  | -Zn:KO vs. +Zn:KO | 0,5477 |  |  |
|  |  |  |  |  |  | +Zn:WT vs. +Zn:KO | 0,9593 |  |  |
| **Suppl. Fig. 6. A** |  | Two-way ANOVA | F interaction (1, 20) = 25,32 | **<0,0001** | ******* | Tukey's post-hoc test |  | 7, 5, 7, 5 |  |
|  |  |  | F treatment (1, 20) = 13,76 | 0,0014 |  | SAL:WT vs. SAL:KO | 0,4211 |  |  |
|  |  |  | F _genotype_ (1, 20) = 9,089 | 0,0068 |  | **SAL:WT vs. ZnCl2:WT** | **<0,0001** |  |  |
|  |  |  |  |  |  | SAL:WT vs. ZnCl2:KO | 0,9601 |  |  |
|  |  |  |  |  |  | SAL:KO vs. ZnCl2:WT | 0,0006 |  |  |
|  |  |  |  |  |  | SAL:KO vs. ZnCl2:KO | 0,7867 |  |  |
|  |  |  |  |  |  | **ZnCl2:WT vs. ZnCl2:KO** | **0,0002** |  |  |
| **Suppl. Fig. 6. B** |  | Two-way ANOVA | F interaction (1, 20) = 0,0007642 | 0,9782 | **n.s** | Tukey's post-hoc test |  | 6, 6, 6, 6 |  |
|  |  |  | F treatment (1, 20) = 0,01910 | 0,6667 |  | +Zn:WT vs. +Zn:KO | 0,5054 |  |  |
|  |  |  | F _genotype_ (1, 20) =3,891 | 0,0625 |  | +Zn:WT vs. -Zn:WT | 0,9874 |  |  |
|  |  |  |  |  |  | +Zn:WT vs. -Zn:KO | 0,3478 |  |  |
|  |  |  |  |  |  | +Zn:KO vs. -Zn:WT | 0,702 |  |  |
|  |  |  |  |  |  | +Zn:KO vs. -Zn:KO | 0,9913 |  |  |
|  |  |  |  |  |  | -Zn:WT vs. -Zn:KO | 0,5285 |  |  |
| **Suppl. Fig. 6. C** |  | Two-way ANOVA | F interaction (1, 14) = 3,838 | 0,0703 | **n.s** | Tukey's post-hoc test |  | 6, 3, 3, 6 |  |
|  |  |  | F treatment (1, 14) = 0,03255 | 0,8594 |  | SAL:WT vs. SAL:KO | 0,8934 |  |  |
|  |  |  | F _genotype_ (1, 14) =0,9249 | 0,3525 |  | SAL:WT vs. ZnCl2:WT | 0,4562 |  |  |
|  |  |  |  |  |  | SAL:WT vs. ZnCl2:KO | 0,9042 |  |  |
|  |  |  |  |  |  | SAL:KO vs. ZnCl2:WT | 0,8956 |  |  |
|  |  |  |  |  |  | SAL:KO vs. ZnCl2:KO | 0,6027 |  |  |
|  |  |  |  |  |  | ZnCl2:WT vs. ZnCl2:KO | 0,2118 |  |  |
| **Suppl. Fig. 6. D** |  | Two-way ANOVA | F interaction (1, 20) = 0,2438 | 0,6269 | **n.s** | Tukey's post-hoc test |  | 6, 4, 7, 7 |  |
|  |  |  | F treatment (1, 20) = 3,526 | 0,0751 |  | +Zn:WT vs. +Zn:KO | <0,0001 |  |  |
|  |  |  | F _genotype_ (1, 20) =713,8 | <0,0001 |  | +Zn:WT vs. -Zn:WT | 0,8057 |  |  |
|  |  |  |  |  |  | +Zn:WT vs. -Zn:KO | <0,0001 |  |  |
|  |  |  |  |  |  | +Zn:KO vs. -Zn:WT | <0,0001 |  |  |
|  |  |  |  |  |  | +Zn:KO vs. -Zn:KO | 0,2765 |  |  |
|  |  |  |  |  |  | -Zn:WT vs. -Zn:KO | <0,0001 |  |  |

**Supplementary table 1.** Statistical table containing the results of the experiments.
