# Supplementary material for: The antidepressant effect of short- and long-term zinc exposition is partly mediated by P2X7 receptors in male mice
Source: Front Pharmacol. 2023 Oct 16;14:1241406. doi: 10.3389/fphar.2023.1241406 (PMC10613712; doi:10.3389/fphar.2023.1241406)
Supplement: Supplementary file 9 [file Table2.DOCX]

Supplementary Material

**The antidepressant effect of short and long-term extracellular zinc is partly mediated by P2X7 receptors**

Bernadett Iring-Varga^1,2^, Mária Baranyi^1^, Flóra Gölöncsér^1^, Pál Tod^1^ and Beáta Sperlágh^1,2^ *

**Correspondence: Beáta Sperlágh**, [sperlagh@koki.hu](mailto:sperlagh@koki.hu)

**Supplementary method 1. Open field test (OFT)**

In order to investigate the effect of zinc on the exploratory behaviour and activity level of mice, an open field test was performed as previously described (Csölle et al., 2013). Before the start of the open field test, the mice were randomly assigned to experimental groups using an Excel protocol. Each 2- to 3-months-old, male animal was individually placed next to the wall of the arena (40 × 40 × 20 cm, covered with an acrylic sheet) and allowed to move freely for 10 min. The behaviour was recorded and analysed using an EthoVision XT 13.0 video-tracking system (Noldus Information Technology, Wageningen, Netherlands). The apparatus was cleaned with 20% ethanol and water after each trial. 30 minutes after the ZnCl_2_ or saline injection, mice were subjected to the test.
