# Supplementary material for: The antidepressant effect of short- and long-term zinc exposition is partly mediated by P2X7 receptors in male mice
Source: Front Pharmacol. 2023 Oct 16;14:1241406. doi: 10.3389/fphar.2023.1241406 (PMC10613712; doi:10.3389/fphar.2023.1241406)
Supplement: Supplementary file 10 [file DataSheet1.pdf]

Supplementary Material

The antidepressant effect of short and long-term extracellular zinc is partly mediated by P2X7 receptors

Bernadett Iring-Varga[1,2], Mária Baranyi[1], Flóra Gölöncsér[1], Pál Tod[1] and Beáta Sperlágh[1,2] *

Correspondence: Beáta Sperlágh, sperlagh@koki.hu

Supplementary Figure 1. Preliminary TST experiments before the start of the study. All tests were performed on young, P2rx7 +/+ male mice. Different doses of $ZnCl_2$ solution were injected into the animals (30 or 60 min before the start) to adjust the treatment: 30 (A) (n=4-5), 10 (B-C) (n=4-5), 3 (D) (n=4-5) and 0,5 (E) (n=4-6) mg/kg. Kaplan-Meier survival analysis performed on experimental groups receiving ZnCl2 solutions (F).

Data are expressed as mean ± S.E.M. Data were analyzed by three-way ANOWA followed by Tukey's test. * $p < 0,05$; ** $p < 0,01$; *** $p < 0,001$.

TST: tail suspension test; WT: wild-type

Supplementary Figure 2. Zinc did not affect locomotor activity and anxiety in mice. (A) Distance travelled by time. (B) Total distance travelled (interaction: $F[1, 20] = 2.938$, $p = 0.1020$). (C) Velocity (interaction: $F[1, 20] = 2.941$, $p = 0.1018$). (D) Cumulative duration in central zone (interaction: $F[1, 20] = 2.254$, $p = 0.1489$). Values are presented as means ± SEM of n = 6 mice/group. Two-way ANOVA (B, C, D) followed by Tukey's multiple comparison post hoc test.

OFT: open field test; WT: wild-type; KO: knock out

Supplementary Figure 3. Preliminary experiment performed on young female +/+ and -/- animals. According to the acute protocol, the mice received 1 mg/kg $ZnCl_2$ solution or SAL i.p. 30 min. before the start of the TST and FST experiments (n=8/group).

Data are expressed as mean ± S.E.M. Data were analyzed by three-way ANOWA followed by Tukey's test. * $p < 0,05$; ** $p < 0,01$; *** $p < 0,001$.

TST: tail suspension test; FST: forced swim test; WT: wild-type; KO: knock out

Supplementary Figure 4. Changes in the weight of young (A) (n=7-10) and elderly (B) (n=9-11) animals receiving chronic treatment as a result of different diets containing zinc.

WT: wild-type; KO: knock out; ZnS: zinc-supplemented diet; ZnD: zinc-deficient diet

Supplementary Figure 5. Monoamine content of Prefrontal Cortex was measured using HPLC technique. Noradrenaline, dopamine, and serotonin levels were plotted in P2rx7 +/+ and P2rx7 -/- elderly, acute (A) (n= 6-7) and chronic treated mice (B) (n= 9-11). Data are expressed as mean ± S.E.M. Data were analyzed by two-way ANOWA followed by Tukey's test. * $p < 0,05$; ** $p < 0,01$; *** $p < 0,001$.

PFC: prefrontal cortex; WT: wild-type; KO: knock out; ZnS: zinc-supplemented diet; ZnD: zinc-deficient diet

Supplementary Figure 6. Examination of prefrontal cortex BDNF protein content by enzyme-linked immunosorbent assay (ELISA) (A-D). Wild type and knock out, young (n= 5-7) (A) and elderly (n=6) (B) mice were injected intraperitoneally with an acute treatment of saline or $ZnCl_2$ (1 mg/kg). P2rx7 +/+ and -/- young (n= 3-6) (C) or elderly mice were fed Zn- controlled diet (n= 4-7) (D). Data are expressed as mean ± S.E.M. Data were analyzed by two-way ANOWA followed by Tukey's test. * p < 0,05; ** p < 0,01; *** p < 0,001.

PFC: prefrontal cortex; WT: wild-type; KO: knock out; ZnS: zinc-supplemented diet; ZnD: zinc-deficient diet

Figure	Monoamine	Statistical Analysis	F-interaction value	p-value		Post hoc test	p-value	n
Fig. 2.A		Two-way ANOVA	F interaction (1, 29) = 2,2780	0,142	n.s	Tukey's post-hoc test		8,7,8,10
			F treatment (1, 29) = 72,77	<0,0001		SAL:WT vs. SAL:KO	<0,0001	
			F genotype (1, 29) = 43,84	<0,0001		SAL:WT vs. 1 mg/kg $ZnCl_2$:WT	<0,0001	
						SAL:WT vs. 1 mg/kg $ZnCl_2$:KO	<0,0001	
						SAL:KO vs. 1 mg/kg $ZnCl_2$:WT	0,7486	
						SAL:KO vs. 1 mg/kg $ZnCl_2$:KO	<0,0001	
						1 mg/kg $ZnCl_2$:WT vs. 1 mg/kg $ZnCl_2$:KO	0,0063	
		Two-way ANOVA	**F interaction (1, 29) = 5,409**	**0,0272**	*	Tukey's post-hoc test		8,7,8,10
			F treatment (1, 29) = 54,78	<0,0001		**SAL:WT vs. SAL:KO**	**<0,0001**	
			F genotype (1, 29) = 42,37	<0,0001		**SAL:WT vs. 1 mg/kg $ZnCl_2$:WT**	**<0,0001**	
						SAL:WT vs. 1 mg/kg $ZnCl_2$:KO	<0,0001	
						SAL:KO vs. 1 mg/kg $ZnCl_2$:WT	0,9319	
						**SAL:KO vs. 1 mg/kg $ZnCl_2$:KO**	**0,0061**	
						**1 mg/kg $ZnCl_2$:WT vs. 1 mg/kg $ZnCl_2$:KO**	**0,0391**	
Fig. 2.B		Two-way ANOVA	F interaction (1, 30) = 0,45990	0,5029	n.s	Tukey's post-hoc test		10,9,6,9
			F treatment (1, 30) = 4,395	0,0446		+Zn:WT vs. +Zn:KO	0,9928	
			F genotype (1, 30) = 0,09657	0,7581		+Zn:WT vs. -Zn:WT	0,1727	
						+Zn:WT vs. -Zn:KO	0,6411	
						+Zn:KO vs. -Zn:WT	0,2789	
						+Zn:KO vs. -Zn:KO	0,7833	
						-Zn:WT vs. -Zn:KO	0,9077	
		Two-way ANOVA	F interaction (1, 31) = 0,6793	0,4161	n.s	Tukey's post-hoc test		10,9,6,9
			F treatment (1, 31) = 90,47	<0,0001		+Zn:WT vs. +Zn:KO	0,1705	
			F genotype (1, 31) = 4,414	0,0439		+Zn:WT vs. -Zn:WT	<0,0001	
						+Zn:WT vs. -Zn:KO	<0,0001	
						+Zn:KO vs. -Zn:WT	<0,0001	
						+Zn:KO vs. -Zn:KO	<0,0001	
						-Zn:WT vs. -Zn:KO	0,8134	
Fig. 2.C		Two-way ANOVA	**F interaction (1, 34) = 6,375**	**0,0164**	*	Tukey's post-hoc test		10, 10, 10, 9
			F treatment (1, 34) = 15,82	0,0003		SAL:WT vs. SAL:KO	0,891	
			F genotype (1, 34) = 2,297	0,1388		**SAL:WT vs. 1 mg/kg $ZnCl_2$:WT**	**0,0003**	
						SAL:WT vs. 1 mg/kg $ZnCl_2$:KO	0,3409	
						SAL:KO vs. 1 mg/kg $ZnCl_2$:WT	0,0018	
						SAL:KO vs. 1 mg/kg $ZnCl_2$:KO	0,7346	
						**1 mg/kg $ZnCl_2$:WT vs. 1 mg/kg $ZnCl_2$:KO**	**0,0348**	

		Two-way ANOVA	F interaction (1, 34) = 0,2846	0,5972	**n.s**	Tukey's post-hoc test		10, 10, 10, 9
			F treatment (1, 34) = 21,81	<0,0001		SAL:WT vs. SAL:KO	0,5571	
			F genotype (1, 34) = 5,760	0,022		SAL:WT vs. 1 mg/kg $ZnCl_2$:WT	0,0043	
						SAL:WT vs. 1 mg/kg $ZnCl_2$:KO	0,4118	
						SAL:KO vs. 1 mg/kg $ZnCl_2$:WT	<0,0001	
						SAL:KO vs. 1 mg/kg $ZnCl_2$:KO	0,0296	
						1 mg/kg $ZnCl_2$:WT vs. 1 mg/kg $ZnCl_2$:KO	0,182	
**Fig. 2.D**		Two-way ANOVA	F interaction (1, 36) = 2,524	0,1209	**n.s**	Tukey's post-hoc test		10,9,6,9
			F treatment (1, 36) = 12,69	0,0011		+Zn:WT vs. +Zn:KO	0,0584	
			F genotype (1, 36) = 5,542	0,0241		+Zn:WT vs. -Zn:WT	0,0039	
						+Zn:WT vs. -Zn:KO	0,001	
						+Zn:KO vs. -Zn:WT	0,9534	
						+Zn:KO vs. -Zn:KO	0,7002	
						-Zn:WT vs. -Zn:KO	0,9947	
		Two-way ANOVA	**F interaction (1, 36) = 78,53**	**<0,0001**	*******	Tukey's post-hoc test		10,9,6,9
			F treatment (1, 36) = 21,81	<0,0001		**+Zn:WT vs. +Zn:KO**	**<0,0001**	
			F genotype (1, 36) = 5,760	0,022		**+Zn:WT vs. -Zn:WT**	**<0,0001**	
						+Zn:WT vs. -Zn:KO	<0,0001	
						+Zn:KO vs. -Zn:WT	0,3792	
						+Zn:KO vs. -Zn:KO	0,4181	
						**-Zn:WT vs. -Zn:KO**	**0,0112**	
**Fig. 3.A**		Two-way ANOVA	F interaction (1, 36) = 3,154	0,0874	**n.s**	Tukey's post-hoc test		8, 8, 6, 8
			F treatment (1, 36) = 43,73	<0,0001		SAL:WT vs. SAL:KO	0,9298	
			$F_{genotype}$ (1, 36) = 18,6	0,0001		SAL:WT vs. 1 mg/kg $ZnCl_2$:WT	<0,0001	
						SAL:WT vs. 1 mg/kg $ZnCl_2$:KO	0,0179	
						SAL:KO vs. 1 mg/kg $ZnCl_2$:WT	<0,0001	
						SAL:KO vs. 1 mg/kg $ZnCl_2$:KO	0,0074	
						1 mg/kg $ZnCl_2$:WT vs. 1 mg/kg $ZnCl_2$:KO	0,0152	
**Fig. 3.B**		Two-way ANOVA	F interaction (1, 18) = 0,03277	0,8584	**n.s**	Tukey's post-hoc test		6, 6, 6, 4
			F treatment (1, 18) = 3,172	0,0918		+Zn:WT vs. +Zn:KO	0,962	
			$F_{genotype}$ (1, 18) =0,6795	0,4206		+Zn:WT vs. -Zn:WT	0,6346	
						+Zn:WT vs. -Zn:KO	0,3294	
						+Zn:KO vs. -Zn:WT	0,8888	
						+Zn:KO vs. -Zn:KO	0,5649	
						-Zn:WT vs. -Zn:KO	0,9054	
**Fig. 3.C**		Two-way ANOVA	**F interaction (1, 26) = 16,58**	**0,0004**	*******	Tukey's post-hoc test		7, 8, 7, 7
			F treatment (1, 26) = 149,8	<0,0001		**SAL:WT vs. SAL:KO**	**0,0137**	
			$F_{genotype}$ (1, 26) =0,3667	0,5495		**SAL:WT vs. 1 mg/kg $ZnCl_2$:WT**	**<0,0001**	
						SAL:WT vs. 1 mg/kg $ZnCl_2$:KO	<0,0001	
						SAL:KO vs. 1 mg/kg $ZnCl_2$:WT	<0,0001	
						**SAL:KO vs. 1 mg/kg $ZnCl_2$:KO**	**<0,0001**	
						1 mg/kg $ZnCl_2$:WT vs. 1 mg/kg $ZnCl_2$:KO	0,0923	
**Fig. 3.D**		Two-way ANOVA	F interaction (1, 29) = 1,736	0,1979	**n.s**	Tukey's post-hoc test		8, 9, 8, 8
			F treatment (1, 29) = 3,189	0,0846		+Zn:WT vs. +Zn:KO	0,9872	
			$F_{genotype}$ (1, 29) =0,7106	0,4061		+Zn:WT vs. -Zn:WT	0,1399	

						+Zn:WT vs. -Zn:KO	0,9121	
						+Zn:KO vs. -Zn:WT	0,256	
						+Zn:KO vs. -Zn:KO	0,9877	
						-Zn:WT vs. -Zn:KO	0,422	
**Fig. 4.A**	*Noradrenaline*	Two-way ANOVA	F interaction (1, 21) = 5,606	0,0276	*	Tukey's post-hoc test		9, 7, 5, 5,
			F treatment (1, 21) = 28,06	<0,0001		**SAL:WT vs. SAL:KO**	**0,025**	
			$F_{genotype}$ (1, 21) =3,144	0,0907		SAL:WT vs. 1 mg/kg $ZnCl_2$:WT	0,1002	
						SAL:WT vs. 1 mg/kg $ZnCl_2$:KO	0,0961	
						SAL:KO vs. 1 mg/kg $ZnCl_2$:WT	0,0003	
						**SAL:KO vs. 1 mg/kg $ZnCl_2$:KO**	**0,0005**	
						**1 mg/kg $ZnCl_2$:WT vs. 1 mg/kg $ZnCl_2$:KO**	**0,9781**	
	Dopamine	Two-way ANOVA	F interaction (1, 21) = 6,451	0,0191	*	Tukey's post-hoc test		9, 7, 5, 5,
			F treatment (1, 21) = 9,634	0,0054		SAL:WT vs. SAL:KO	0,9177	
			$F_{genotype}$ (1, 21) =2,850	0,1061		SAL:WT vs. 1 mg/kg $ZnCl_2$:WT	0,9649	
						SAL:WT vs. 1 mg/kg $ZnCl_2$:KO	0,015	
						SAL:KO vs. 1 mg/kg $ZnCl_2$:WT	0,7431	
						**SAL:KO vs. 1 mg/kg $ZnCl_2$:KO**	**0,0099**	
						**1 mg/kg $ZnCl_2$:WT vs. 1 mg/kg $ZnCl_2$:KO**	**0,0458**	
	Serotonin	Two-way ANOVA	F interaction (1, 22) = 0,03745	0,8483	**n.s**	Tukey's post-hoc test		9, 8, 5, 5,
			F treatment (1, 22) = 12,81	0,0017		SAL:WT vs. SAL:KO	0,9085	
			$F_{genotype}$ (1, 22) = 1,191	0,2869		SAL:WT vs. 1 mg/kg $ZnCl_2$:WT	0,0193	
						SAL:WT vs. 1 mg/kg $ZnCl_2$:KO	0,3401	
						SAL:KO vs. 1 mg/kg $ZnCl_2$:WT	0,0129	
						SAL:KO vs. 1 mg/kg $ZnCl_2$:KO	0,0097	
						1 mg/kg $ZnCl_2$:WT vs. 1 mg/kg $ZnCl_2$:KO	0,8206	
**Fig. 4.B**	*Noradrenaline*	Two-way ANOVA	F interaction (1, 22) = 0,4623	0,5036	**n.s**	Tukey's post-hoc test		6, 7, 6, 7
			F treatment (1, 22) = 1,570	0,2234		+Zn:WT vs. +Zn:KO	0,9969	
			$F_{genotype}$ (1, 22) = 0,1449	0,7071		+Zn:WT vs. -Zn:WT	0,5324	
						+Zn:WT vs. -Zn:KO	0,9256	
						+Zn:KO vs. -Zn:WT	0,6604	
						+Zn:KO vs. -Zn:KO	0,977	
						-Zn:WT vs. -Zn:KO	0,8624	
	Dopamine	Two-way ANOVA	F interaction (1, 22) = 0,3855	0,541	**n.s**	Tukey's post-hoc test		6, 7, 6, 7
			F treatment (1, 22) = 1,988	0,1726		+Zn:WT vs. +Zn:KO	0,7677	
			$F_{genotype}$ (1, 22) = 0,6441	0,4308		+Zn:WT vs. -Zn:WT	0,9434	
						+Zn:WT vs. -Zn:KO	0,9728	
						+Zn:KO vs. -Zn:WT	0,4184	
						+Zn:KO vs. -Zn:KO	0,4914	
						-Zn:WT vs. -Zn:KO	0,9991	
	Serotonin	Two-way ANOVA	**F interaction (1, 23) = 21,87**	**0,0001**	*****	Tukey's post-hoc test		7, 7, 6, 7
			F treatment (1, 23) = 3,423	0,0772		**+Zn:WT vs. +Zn:KO**	**0,0002**	
			$F_{genotype}$ (1, 23) = 7,859	0,0101		**+Zn:WT vs. -Zn:WT**	**0,0005**	
						+Zn:WT vs. -Zn:KO	0,0135	
						+Zn:KO vs. -Zn:WT	0,9106	
						+Zn:KO vs. -Zn:KO	0,2321	

						-Zn:WT vs. -Zn:KO	0,5409	
**Fig. 4.C**	*Noradrenaline*	Two-way ANOVA	F interaction (1, 27) = 0,005913	0,9393	**n.s**	Tukey's post-hoc test		9, 9, 6, 7
			F treatment (1, 27) = 0,6902	0,4134		SAL:WT vs. SAL:KO	0,997	
			$F_{genotype}$ (1, 27) = 0,1353	0,7159		SAL:WT vs. 1 mg/kg $ZnCl_2$:WT	0,9363	
						SAL:WT vs. 1 mg/kg $ZnCl_2$:KO	0,8216	
						SAL:KO vs. 1 mg/kg $ZnCl_2$:WT	0,9884	
						SAL:KO vs. 1 mg/kg $ZnCl_2$:KO	0,9328	
						1 mg/kg $ZnCl_2$:WT vs. 1 mg/kg $ZnCl_2$:KO	0,9882	
	Dopamine	Two-way ANOVA	F interaction (1, 27) = 0,2542	0,6182	**n.s**	Tukey's post-hoc test		9, 9, 6, 7
			F treatment (1, 27) = 1,203	0,2823		SAL:WT vs. SAL:KO	0,9663	
			$F_{genotype}$ (1, 27) = 0,02760	0,8693		SAL:WT vs. 1 mg/kg $ZnCl_2$:WT	0,6086	
						SAL:WT vs. 1 mg/kg $ZnCl_2$:KO	0,7977	
						SAL:KO vs. 1 mg/kg $ZnCl_2$:WT	0,9167	
						SAL:KO vs. 1 mg/kg $ZnCl_2$:KO	0,9797	
						1 mg/kg $ZnCl_2$:WT vs. 1 mg/kg $ZnCl_2$:KO	0,9947	
	Serotonin	Two-way ANOVA	F interaction (1, 27) = 0,7987	0,3794	**n.s**	Tukey's post-hoc test		9, 9, 6, 7
			F treatment (1, 27) = 8,431	0,0073		SAL:WT vs. SAL:KO	0,6214	
			$F_{genotype}$ (1, 27) =0,7502	0,394		SAL:WT vs. 1 mg/kg $ZnCl_2$:WT	0,032	
						SAL:WT vs. 1 mg/kg $ZnCl_2$:KO	0,051	
						SAL:KO vs. 1 mg/kg $ZnCl_2$:WT	0,5045	
						SAL:KO vs. 1 mg/kg $ZnCl_2$:KO	0,5603	
						1 mg/kg $ZnCl_2$:WT vs. 1 mg/kg $ZnCl_2$:KO	>0,9999	
**Fig. 4.D**	*Noradrenaline*	Two-way ANOVA	F interaction (1, 36) = 2,628	0,1137	**n.s**	Tukey's post-hoc test		10, 11, 9, 10
			F treatment (1, 36) = 25,19	<0,0001		-Zn:WT vs. -Zn:KO	0,0014	
			$F_{genotype}$ (1, 36) =51,88	<0,0001		-Zn:WT vs. +Zn:WT	0,0001	
						-Zn:WT vs. +Zn:KO	0,4246	
						-Zn:KO vs. +Zn:WT	<0,0001	
						-Zn:KO vs. +Zn:KO	0,1069	
						+Zn:WT vs. +Zn:KO	<0,0001	
	Dopamine	Two-way ANOVA	F interaction (1, 36) = 1,475	0,2325	**n.s**	Tukey's post-hoc test		10, 11, 9, 10
			F treatment (1, 36) = 13,27	0,0008		-Zn:WT vs. -Zn:KO	0,5155	
			$F_{genotype}$ (1, 36) =9,771	0,0035		-Zn:WT vs. +Zn:WT	0,0062	
						-Zn:WT vs. +Zn:KO	0,9831	
						-Zn:KO vs. +Zn:WT	0,0002	
						-Zn:KO vs. +Zn:KO	0,351	
						+Zn:WT vs. +Zn:KO	0,0244	
	Serotonin	Two-way ANOVA	F interaction (1, 36) = 0,5137	0,4782	**n.s**	Tukey's post-hoc test		10, 11, 9, 10
			F treatment (1, 36) = 24,25	<0,0001		-Zn:WT vs. -Zn:KO	<0,0001	
			$F_{genotype}$ (1, 36) =41,18	<0,0001		-Zn:WT vs. +Zn:WT	0,0211	
						-Zn:WT vs. +Zn:KO	0,7199	
						-Zn:KO vs. +Zn:WT	<0,0001	
						-Zn:KO vs. +Zn:KO	0,0022	
						+Zn:WT vs. +Zn:KO	0,002	
**Fig. 5.A**		Two-way ANOVA	F interaction (1, 25) = 0,8278	0,3716	**n.s**	Tukey's post-hoc test		11, 7, 7, 5
			F treatment (1, 25) = 0,03456	0,854		SAL:WT vs. SAL:KO	0,0105	
			$F_{genotype}$ (1, 25) = 12,33	0,0017		SAL:WT vs. ZnCl2:WT	0,8297	

						SAL:WT vs. ZnCl2:KO	0,0713	
						SAL:KO vs. ZnCl2:WT	0,1071	
						SAL:KO vs. ZnCl2:KO	0,9643	
						ZnCl2:WT vs. ZnCl2:KO	0,3448	
**Fig. 5.B**		Two-way ANOVA	F interaction (1, 20) = 3,856	0,0636	**n.s**	Tukey's post-hoc test		6, 6, 6, 6
			F treatment (1, 20) = 0,0041	0,9498		+Zn:WT vs. +Zn:KO	0,0159	
			$F_{genotype}$ (1, 20) =7,597	0,0122		+Zn:WT vs. -Zn:WT	0,4941	
						+Zn:WT vs. -Zn:KO	0,2233	
						+Zn:KO vs. -Zn:WT	0,258	
						+Zn:KO vs. -Zn:KO	0,5475	
						-Zn:WT vs. -Zn:KO	0,9426	
**Fig. 5.C**		Two-way ANOVA	F interaction (1, 26) = 2,802	0,1061	**n.s**	Tukey's post-hoc test		11, 7, 6, 6
			F treatment (1, 26) = 0,0508	0,8234		SAL:WT vs. SAL:KO	0,0966	
			$F_{genotype}$ (1, 26) =2,08	0,1608		SAL:WT vs. ZnCl2:WT	0,5051	
						SAL:WT vs. ZnCl2:KO	0,6089	
						SAL:KO vs. ZnCl2:WT	0,8424	
						SAL:KO vs. ZnCl2:KO	0,7618	
						ZnCl2:WT vs. ZnCl2:KO	0,9988	
**Fig. 5.D**		Two-way ANOVA	F interaction (1, 35) = 1,487	0,2308	**n.s**	Tukey's post-hoc test		10, 11, 9, 9
			F treatment (1, 35) = 10,65	0,0025		+Zn:WT vs. +Zn:KO	<0,0001	
			$F_{genotype}$ (1, 35) =71,22	<0,0001		+Zn:WT vs. -Zn:WT	0,0115	
						+Zn:WT vs. -Zn:KO	0,0049	
						+Zn:KO vs. -Zn:WT	<0,0001	
						+Zn:KO vs. -Zn:KO	0,5117	
						-Zn:WT vs. -Zn:KO	<0,0001	
**Figure**		**Statistical Analysis**	**One- or two-tailed P value?**	**p-value**				**n**
**Suppl fig.1.A**		Unpaired t-test	Two-tailed	**0,026**	*****			4, 5
**Suppl fig.1.B**		Unpaired t-test	Two-tailed	**0,0003**	*******			5, 4
**Suppl fig.1.C**		Unpaired t-test	Two-tailed	**0,0062**	******			4, 4
**Suppl fig.1.D**		Unpaired t-test	Two-tailed	0,2712	**n.s**			4, 5
**Suppl fig.1.E**		Unpaired t-test	Two-tailed	0,1953	**n.s**			4, 5
**Figure**	**Experiment**	**Statistical Analysis**	**F-interaction value**	**p-value**		**Post hoc test**	**p-value**	**n**
**Suppl. Fig. 2. B**		Two-way ANOVA	F interaction (1, 20) = 2,938	0,102	**n.s**	Tukey's post-hoc test		6, 6, 6, 6
			F treatment (1, 20) = 0,1017	0,7531		SAL:WT vs. SAL:KO	0,6982	
			$F_{genotype}$ (1, 20) = 0,02873	0,8671		SAL:WT vs. ZnCl2:WT	0,4918	
						SAL:WT vs. ZnCl2:KO	0,9996	
						SAL:KO vs. ZnCl2:WT	0,9854	
						SAL:KO vs. ZnCl2:KO	0,7586	
						ZnCl2:WT vs. ZnCl2:KO	0,5543	
		Two-way ANOVA	F interaction (1, 20) = 2,941	0,1018	**n.s**	Tukey's post-hoc test		6, 6, 6, 6

**Suppl. Fig. 2. C**			F treatment (1, 20) = 0,1025	0,7522		SAL:WT vs. SAL:KO	0,6968	
			$F_{genotype}$ (1, 20) =0,02789	0,869		SAL:WT vs. ZnCl2:WT	0,4909	
						SAL:WT vs. ZnCl2:KO	0,9995	
						SAL:KO vs. ZnCl2:WT	0,9855	
						SAL:KO vs. ZnCl2:KO	0,7588	
						ZnCl2:WT vs. ZnCl2:KO	0,555	
**Suppl. Fig. 2. D**		Two-way ANOVA	F interaction (1, 20) = 2,254	0,1489	**n.s**	Tukey's post-hoc test		6, 6, 6, 6
			F treatment (1, 20) = 0,0002328	0,988		SAL:WT vs. SAL:KO	0,8094	
			$F_{genotype}$ (1, 20) =0,05810	0,812		SAL:WT vs. ZnCl2:WT	0,7222	
						SAL:WT vs. ZnCl2:KO	0,9978	
						SAL:KO vs. ZnCl2:WT	0,9985	
						SAL:KO vs. ZnCl2:KO	0,7097	
						ZnCl2:WT vs. ZnCl2:KO	0,6145	
**Suppl fig. 3**	TST	Two-way ANOVA	F interaction (1, 28) = 0,1220	0,7294	**n.s**	Tukey's post-hoc test		8, 8, 8, 8
			F treatment (1, 28) = 15,94	0,0004		SAL:WT vs. SAL:KO	0,9964	
			$F_{genotype}$ (1, 28) = 0,002125	0,9636		SAL:WT vs. 1 mg/kg $ZnCl_2$:WT	0,023	
						SAL:WT vs. 1 mg/kg $ZnCl_2$:KO	0,0438	
						SAL:KO vs. 1 mg/kg $ZnCl_2$:WT	0,0378	
						SAL:KO vs. 1 mg/kg $ZnCl_2$:KO	0,0698	
						1 mg/kg $ZnCl_2$:WT vs. 1 mg/kg $ZnCl_2$:KO	0,9922	
	FST	Two-way ANOVA	F interaction (1, 28) = 0,01623	0,8995	**n.s**	Tukey's post-hoc test		8, 8, 8, 8
			F treatment (1, 28) = 0,5411	0,4681		SAL:WT vs. SAL:KO	0,7879	
			$F_{genotype}$ (1, 28) =1,419	0,2436		SAL:WT vs. 1 mg/kg $ZnCl_2$:WT	0,9728	
						SAL:WT vs. 1 mg/kg $ZnCl_2$:KO	0,9882	
						SAL:KO vs. 1 mg/kg $ZnCl_2$:WT	0,5325	
						SAL:KO vs. 1 mg/kg $ZnCl_2$:KO	0,928	
						1 mg/kg $ZnCl_2$:WT vs. 1 mg/kg $ZnCl_2$:KO	0,875	
**Suppl fig. 5.A**	*Noradrenaline*	Two-way ANOVA	F interaction (1, 27) = 0,0005260	0,9819	**n.s**	Tukey's post-hoc test		9, 9, 6, 7
			F treatment (1, 27) = 1,963	0,1726		SAL:WT vs. SAL:KO	0,9326	
			$F_{genotype}$ (1, 27) =0,7018	0,4095		SAL:WT vs. 1 mg/kg ZnCl2:WT	0,6917	
						SAL:WT vs. 1 mg/kg ZnCl2:KO	0,3849	
						SAL:KO vs. 1 mg/kg ZnCl2:WT	0,9795	
						SAL:KO vs. 1 mg/kg ZnCl2:KO	0,8032	
						1 mg/kg ZnCl2:WT vs. 1 mg/kg ZnCl2:KO	0,9344	
	Dopamine	Two-way ANOVA	**F interaction (1, 27) = 4,464**	**0,044**	*	Tukey's post-hoc test		9, 9, 6, 7
			F treatment (1, 27) = 1,592	0,2179		SAL:WT vs. SAL:KO	0,9414	
			$F_{genotype}$ (1, 27) =1,678	0,2062		SAL:WT vs. 1 mg/kg ZnCl2:WT	0,0657	
						SAL:WT vs. 1 mg/kg ZnCl2:KO	>0,9999	
						SAL:KO vs. 1 mg/kg ZnCl2:WT	0,3098	
						SAL:KO vs. 1 mg/kg ZnCl2:KO	0,9436	
						1 mg/kg ZnCl2:WT vs. 1 mg/kg ZnCl2:KO	0,0886	
	Serotonin	Two-way ANOVA	F interaction (1, 27) = 0,03403	0,855	**n.s**	Tukey's post-hoc test		9, 9, 6, 7
			F treatment (1, 27) = 13,63	0,001		SAL:WT vs. SAL:KO	0,9961	
			$F_{genotype}$ (1, 27) =0,2527	0,6192		SAL:WT vs. 1 mg/kg ZnCl2:WT	0,0278	
						SAL:WT vs. 1 mg/kg ZnCl2:KO	0,1214	

						SAL:KO vs. 1 mg/kg ZnCl2:WT	0,0346	
						SAL:KO vs. 1 mg/kg ZnCl2:KO	0,1234	
						1 mg/kg ZnCl2:WT vs. 1 mg/kg ZnCl2:KO	0,959	
**Suppl fig. 5.B**	*Noradrenaline*	Two-way ANOVA	**F interaction (1, 36) = 58,75**	**<0,0001**	***	Tukey's post-hoc test		10, 11, 9, 10
			F treatment (1, 36) = 21,41	<0,0001		**-Zn:WT vs. -Zn:KO**	**<0,0001**	
			$F_{genotype}$ (1, 36) =32,52	<0,0001		**-Zn:WT vs. +Zn:WT**	**<0,0001**	
						-Zn:WT vs. +Zn:KO	<0,0001	
						-Zn:KO vs. +Zn:WT	0,8708	
						-Zn:KO vs. +Zn:KO	0,1738	
						+Zn:WT vs. +Zn:KO	0,5356	
	Dopamine	Two-way ANOVA	F interaction (1, 35) = 1,424	0,2408	n.s	Tukey's post-hoc test		10, 10, 9, 10
			F treatment (1, 35) = 1,061e-005	0,9974		-Zn:WT vs. -Zn:KO	0,0004	
			$F_{genotype}$ (1, 35) =25,86	<0,0001		-Zn:WT vs. +Zn:WT	0,8287	
						-Zn:WT vs. +Zn:KO	0,006	
						-Zn:KO vs. +Zn:WT	0,0045	
						-Zn:KO vs. +Zn:KO	0,8376	
						+Zn:WT vs. +Zn:KO	0,0479	
	Serotonin	Two-way ANOVA	**F interaction (1, 36) = 34,44**	**<0,0001**	***	Tukey's post-hoc test		10, 11, 9, 10
			F treatment (1, 36) = 15,48	0,0004		**-Zn:WT vs. -Zn:KO**	**<0,0001**	
			$F_{genotype}$ (1, 36) =43,40	<0,0001		**-Zn:WT vs. +Zn:WT**	**<0,0001**	
						-Zn:WT vs. +Zn:KO	<0,0001	
						-Zn:KO vs. +Zn:WT	0,254	
						-Zn:KO vs. +Zn:KO	0,5477	
						+Zn:WT vs. +Zn:KO	0,9593	
**Suppl. Fig. 6. A**		Two-way ANOVA	F interaction (1, 20) = 25,32	**<0,0001**	***	Tukey's post-hoc test		7, 5, 7, 5
			F treatment (1, 20) = 13,76	0,0014		SAL:WT vs. SAL:KO	0,4211	
			$F_{genotype}$ (1, 20) = 9,089	0,0068		**SAL:WT vs. ZnCl2:WT**	**<0,0001**	
						SAL:WT vs. ZnCl2:KO	0,9601	
						SAL:KO vs. ZnCl2:WT	0,0006	
						SAL:KO vs. ZnCl2:KO	0,7867	
						**ZnCl2:WT vs. ZnCl2:KO**	**0,0002**	
**Suppl. Fig. 6. B**		Two-way ANOVA	F interaction (1, 20) = 0,0007642	0,9782	n.s	Tukey's post-hoc test		6, 6, 6, 6
			F treatment (1, 20) = 0,01910	0,6667		+Zn:WT vs. +Zn:KO	0,5054	
			$F_{genotype}$ (1, 20) =3,891	0,0625		+Zn:WT vs. -Zn:WT	0,9874	
						+Zn:WT vs. -Zn:KO	0,3478	
						+Zn:KO vs. -Zn:WT	0,702	
						+Zn:KO vs. -Zn:KO	0,9913	
						-Zn:WT vs. -Zn:KO	0,5285	
**Suppl. Fig. 6. C**		Two-way ANOVA	F interaction (1, 14) = 3,838	0,0703	n.s	Tukey's post-hoc test		6, 3, 3, 6
			F treatment (1, 14) = 0,03255	0,8594		SAL:WT vs. SAL:KO	0,8934	
			$F_{genotype}$ (1, 14) =0,9249	0,3525		SAL:WT vs. ZnCl2:WT	0,4562	
						SAL:WT vs. ZnCl2:KO	0,9042	
						SAL:KO vs. ZnCl2:WT	0,8956	
						SAL:KO vs. ZnCl2:KO	0,6027	
						ZnCl2:WT vs. ZnCl2:KO	0,2118	

**Suppl. Fig. 6. D**		Two-way ANOVA	F interaction (1, 20) = 0,2438	0,6269	**n.s**	Tukey's post-hoc test		6, 4, 7, 7
			F treatment (1, 20) = 3,526	0,0751		+Zn:WT vs. +Zn:KO	<0,0001	
			$F_{genotype}$ (1, 20) =713,8	<0,0001		+Zn:WT vs. -Zn:WT	0,8057	
						+Zn:WT vs. -Zn:KO	<0,0001	
						+Zn:KO vs. -Zn:WT	<0,0001	
						+Zn:KO vs. -Zn:KO	0,2765	
						-Zn:WT vs. -Zn:KO	<0,0001	

Supplementary table 1. Statistical table containing the results of the experiments.
